# Chitosan‐based nanoscale systems for doxorubicin delivery: Exploring biomedical application in cancer therapy

**DOI:** 10.1002/btm2.10325

**Published:** 2022-09-13

**Authors:** Milad Ashrafizadeh, Kiavash Hushmandi, Sepideh Mirzaei, Saied Bokaie, Ashkan Bigham, Pooyan Makvandi, Navid Rabiee, Vijay Kumar Thakur, Alan Prem Kumar, Esmaeel Sharifi, Rajender S. Varma, Amir Reza Aref, Marcin Wojnilowicz, Ali Zarrabi, Hassan Karimi‐Maleh, Nicolas H. Voelcker, Ebrahim Mostafavi, Gorka Orive

**Affiliations:** ^1^ Faculty of Engineering and Natural Sciences Sabanci University, Üniversite Caddesi Tuzla, Istanbul Turkey; ^2^ Department of Food Hygiene and Quality Control, Division of Epidemiology, Faculty of Veterinary Medicine University of Tehran Tehran Iran; ^3^ Department of Biology, Faculty of Science Islamic Azad University, Science and Research Branch Tehran Iran; ^4^ Institute of Polymers, Composites and Biomaterials ‐ National Research Council (IPCB‐CNR) Naples Italy; ^5^ Istituto Italiano di Tecnologia, Center for Materials Interfaces Pontedera, Pisa Italy; ^6^ School of Engineering, Macquarie University Sydney New South Wales Australia; ^7^ School of Engineering University of Petroleum & Energy Studies (UPES) Dehradun Uttarakhand India; ^8^ Biorefining and Advanced Materials Research Center, Scotland's Rural College (SRUC) Edinburgh UK; ^9^ NUS Centre for Cancer Research (N2CR) Yong Loo Lin School of Medicine, National University of Singapore Singapore Singapore; ^10^ Department of Pharmacology Yong Loo Lin School of Medicine, National University of Singapore Kent Ridge Singapore; ^11^ Department of Tissue Engineering and Biomaterials School of Advanced Medical Sciences and Technologies, Hamadan University of Medical Sciences Hamadan Iran; ^12^ Regional Center of Advanced Technologies and Materials Czech Advanced Technology and Research Institute, Palacky University Olomouc Czech Republic; ^13^ Belfer Center for Applied Cancer Science, Dana‐Farber Cancer Institute, Harvard Medical School Boston Massachusetts USA; ^14^ Xsphera Biosciences Inc. Boston Massachusetts USA; ^15^ Commonwealth Scientific and Industrial Research Organisation (CSIRO) Manufacturing Clayton Victoria Australia; ^16^ Monash Institute of Pharmaceutical Sciences Parkville Victoria Australia; ^17^ Department of Biomedical Engineering, Faculty of Engineering and Natural Sciences Istinye University Istanbul Turkey; ^18^ School of Resources and Environment, University of Electronic Science and Technology of China Chengdu PR China; ^19^ Department of Chemical Engineering Quchan University of Technology Quchan Iran; ^20^ Department of Chemical Sciences, University of Johannesburg, Doornfontein Campus Johannesburg South Africa; ^21^ Melbourne Centre for Nanofabrication Victorian Node of the Australian National Fabrication Facility Clayton Victoria Australia; ^22^ Stanford Cardiovascular Institute, Stanford University School of Medicine Stanford California USA; ^23^ Department of Medicine Stanford University School of Medicine Stanford California USA; ^24^ NanoBioCel Research Group, School of Pharmacy University of the Basque Country (UPV/EHU) Vitoria‐Gasteiz Spain; ^25^ University Institute for Regenerative Medicine and Oral Implantology–UIRMI(UPV/EHU‐Fundación Eduardo Anitua) Vitoria‐Gasteiz Spain; ^26^ Bioaraba, NanoBioCel Research Group Vitoria‐Gasteiz Spain; ^27^ Singapore Eye Research Institute Singapore

**Keywords:** chitosan, drug resistance, gene therapy, stimuli‐responsive nanocarriers, synergistic therapy

## Abstract

Green chemistry has been a growing multidisciplinary field in recent years showing great promise in biomedical applications, especially for cancer therapy. Chitosan (CS) is an abundant biopolymer derived from chitin and is present in insects and fungi. This polysaccharide has favorable characteristics, including biocompatibility, biodegradability, and ease of modification by enzymes and chemicals. CS‐based nanoparticles (CS‐NPs) have shown potential in the treatment of cancer and other diseases, affording targeted delivery and overcoming drug resistance. The current review emphasizes on the application of CS‐NPs for the delivery of a chemotherapeutic agent, doxorubicin (DOX), in cancer therapy as they promote internalization of DOX in cancer cells and prevent the activity of P‐glycoprotein (P‐gp) to reverse drug resistance. These nanoarchitectures can provide co‐delivery of DOX with antitumor agents such as curcumin and cisplatin to induce synergistic cancer therapy. Furthermore, co‐loading of DOX with siRNA, shRNA, and miRNA can suppress tumor progression and provide chemosensitivity. Various nanostructures, including lipid‐, carbon‐, polymeric‐ and metal‐based nanoparticles, are modifiable with CS for DOX delivery, while functionalization of CS‐NPs with ligands such as hyaluronic acid promotes selectivity toward tumor cells and prevents DOX resistance. The CS‐NPs demonstrate high encapsulation efficiency and due to protonation of amine groups of CS, pH‐sensitive release of DOX can occur. Furthermore, redox‐ and light‐responsive CS‐NPs have been prepared for DOX delivery in cancer treatment. Leveraging these characteristics and in view of the biocompatibility of CS‐NPs, we expect to soon see significant progress towards clinical translation.

**Abbreviations**
AA: acrylic acid
ABC: ATP‐binding cassette
AGO: amine‐functionalized GO
BSA: bovine serum albumin
CHOL: cholesterol
CMC: carboxymethyl CS
CSO: CS oligosaccharide
CXB: celecoxib
DCA: deoxycholic acid
DOX: doxorubicin
FA: folic acid
GA: glycyrrhetinic acid
GO: graphene oxide
GSH: glutathione
HA: hyaluronic acid
HSPC: hydrogenated soy phosphatidyl choline
IA: itaconic acid
miRNA: microRNA
M‐MSNs: magnetic mesoporous silica nanoparticles
MOFs: metal organic frameworks
OA: oleanolic acid
P‐gp: P‐glycoprotein
PHA: pheophorbide A
PTX: paclitaxel
RAPA: rapamycin
ROS: reactive oxygen species
SA: stearic acid
shRNA: short hairpin RNA
siRNA: small interfering RNA
SOC: *N*‐succinyl‐*N*′‐octyl chitosan

## 1 INTRODUCTION

Cancer treatment requires development of therapeutic strategies for minimizing growth and migration of tumor cells to improve overall survival of patients. For exerting such activities, antitumor compounds should be effectively internalized by cancer cells and induce a therapeutic effect at the cellular level by affecting the molecular pathways and mechanisms responsible for organelle organization and function, such as mitochondria and endoplasmic reticulum.^1^, ^2^, ^3^, ^4^, ^5^, ^6^, ^7^, ^8^ Due to advancement in the fields of medicinal chemistry, various antitumor compounds, namely cisplatin, paclitaxel, docetaxel, and doxorubicin (DOX) among others, have been developed in cancer therapy.^9^, ^10^ Chemotherapy is currently a first‐line option for the treatment of cancer patients to eradicate tumor progression and improve prognosis. Furthermore, chemotherapy is preferred to surgery, as it is a noninvasive strategy in cancer treatment. However, some of the cancers are inherently resistant to chemotherapy, or attained drug resistance during the treatment affecting antitumor agent or its target. As chemoresistance threatens the life of many people around the world, there have been incessant efforts in understanding underlying factors in this process. The drug resistance is a multifactorial condition and each factor can independently participate in decreasing cytotoxicity of antitumor agent. The enhanced drug efflux, diminution in drug uptake, mutation, drug inactivation, apoptosis machinery impairment, signaling networks (upregulation of tumor‐promoting factors and downregulation of tumor‐suppressor), and phenotype switching are the mechanisms that can lead to cancer drug resistance.^11^, ^12^, ^13^, ^14^ Given the importance of drug resistance in chemotherapy failure, scientists have followed some strategies for overcoming this condition by applying nanostructures that improve drug delivery potential, enhance intracellular accumulation, and provide targeted delivery and co‐delivery with other antitumor agents or nucleic acid therapeutics.^15^, ^16^

The aim of present review is to discuss the role of chitosan (CS) for the delivery of DOX as one of the most well‐known chemotherapeutic agents in cancer therapy and introducing CS chemistry, structure, and potential applications in medicine. The function of DOX in cancer suppression, factors responsible for its resistance and role of nanoparticles in reversing DOX resistance are discussed with emphasis on CS‐based nanostructures for its delivery; pH‐ and redox‐sensitive assorted CS nanoparticles are highlighted including their use for co‐delivery of DOX with antitumor agents and nucleic acid therapeutics. Finally, the modification of various nanoparticles and appropriate solutions for their clinical applications are described to shed a light on the deployment of these nanostructures for cancer chemotherapy.

## 2 CHITOSAN: CHEMISTRY AND BIOMEDICAL APPLICATION

The green technology is one of the newest and most recent approaches for the development of nanopharmaceuticals in treatment of diseases. The green chemistry approach utilizes compounds and agents derived from nature for synthesis and modification of nanocarriers to improve their characteristics and make them better options for disease treatment. In this strategy, the hazardous material application is avoided and in turn, safe, biorenewable and biocompatible agents isolated from nature are utilized to develop nanoparticles. Since green‐based nanocarriers demonstrate good safety profile and biocompatibility, the way for their clinical application is paved. The delivery of drugs and nucleic acid therapeutics is essential in cancer therapy due to their low accumulation at tumor site and emergence of drug resistance; hence, green synthesis or green modification of nanoparticles can be beneficial in this case for improving efficacy in cancer therapy.^17^, ^18^, ^19^ In the present review, our aim is to highlight greener modifications of nanoparticles with CS as a natural compound to show its potential for cancer chemotherapy and possible clinical applications in the near future.

**FIGURE 1:**
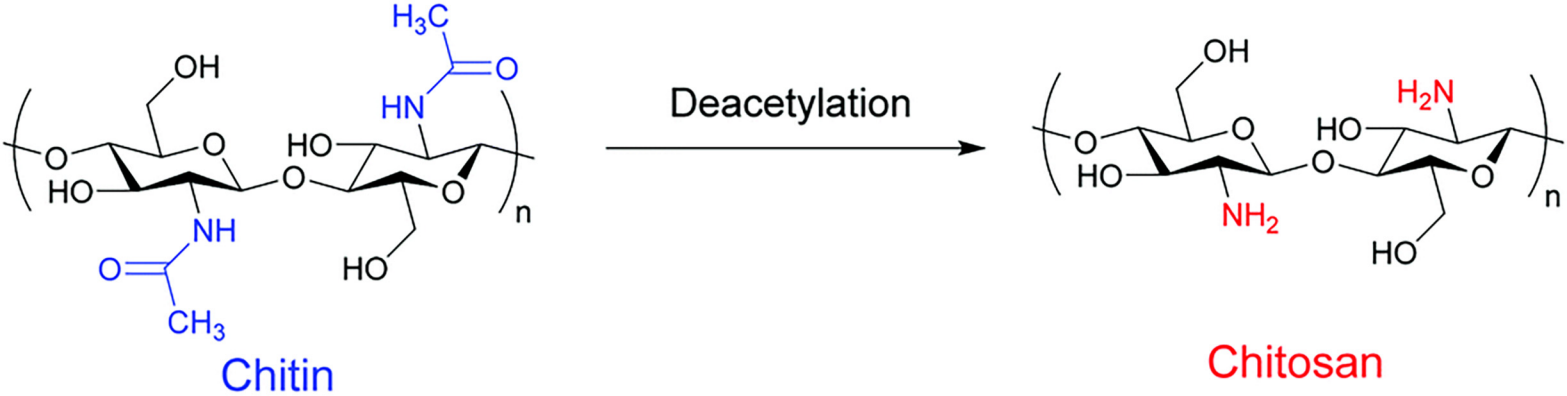
Chemical structures of chitin and CS via deacetylation.*Source*: Reprinted with permission from Ref 21

After cellulose, chitin is the most abundant natural polymer^20^ and CS is derived from chitin (Figure 1),^22^ an essential component comprising shells of insects, crustaceans, and cell walls of fungi.^23^, ^24^, ^25^ The annual production of chitin is estimated to be 10–100 Gt and the commercialized chitin/CS can result from seafood waste in which α‐ and β‐chitins are derived from shells of crab and shrimp, while CS is prepared by deacetylation of chitin.^23^, ^24^, ^26^, ^27^, ^28^ The amount of deacetylation seems to be more than 60% in commercialized CS where Japan is considered as the major producer of CS. Based on the estimates, the value of CS market has been ~6.8 B$ in 2019.^20^ The unique chemical structure of CS has made it a suitable option for biomedical and engineering applications. The most important feature of CS is its great solubility in aqueous solution due to the presence of amino groups at C2 position. In aqueous acidic solvents, CS undergoes protonation to generate NH_3_^+^ that is beneficial in the design of nanoarchitectures and their synthesis via bottom‐up approach. Importantly, amino and acetylamino groups in CS are main sources of nitrogen for generating fertilizers and N‐doped carbon materials for deployment as catalyst.^20^, ^29^

The application of CS in industry has demonstrated potential in reducing environmental pollution as a biodegradable and renewable abundant material that should not be discarded in to scarce landfills. The aim of green chemistry is to limit industrial production of hazardous compounds and prevent destructive impacts, both short‐term and long‐term, on ecosystem.^30^, ^31^, ^32^ Besides, green chemistry is beneficial in decreasing energy consumption and substituting conventional solvents with newer options that are renewable and demonstrate low destructive impact on environment.^33^ The precursor of CS, chitin is made of up to 3000 repeating units comprising *N*‐acetyl‐d‐glucosamine^34^ that are interconnected via β(1 → 4) glycosidic bonds. The chitin exhibits high similarity to cellulose in terms of chemical structure with the difference that hydroxyl group at position C2 is substituted by acetamido group.^35^ There are various kinds of chitin including α, β, and γ, which show variation in hydration, size, and number of chains^36^ and are present in various structures and sources. For instance, α‐chitin is found in shells and cell walls, β‐chitin is present in endocycleton of squid pens^36^, ^37^ and γ‐chitin is observed in stomach lining of squid and cuttlefish.^36^

There are two saccharides, namely, *N*‐acetyl‐d‐glucosamine and β 1–4 d‐glucosamine in the CS structure and during the deacetylation of chitin, *N*‐acetyl‐d‐glucosamine monomers are transformed into d‐glucosamine to generate CS. The LD^50^ of CS is 16 g/kg body weight and it shows a great safety profile. The various kinds of CS are categorized based on molecular weight and deacetylation degree^38^; CS is a polycation and its charge density is determined by pH and deacetylation degree including the solubility aspects. The CS oligomers display solubility in acidic and basic media but with increase in its molecular weight, it is only soluble in acidic media even with a higher deacetylation degree. Consequently, significant efforts have been made in synthesizing CS derivatives that are soluble under neutral and basic pH conditions by altering acetylation, polymerization, and quaternization^39^; pKa value is suggested to be 6.5 and protonation of —NH_2_ groups provides CS solubility in acidic media^40^ as has been affirmed that protonation of 50% of amine groups leads to CS solubility.^41^ The molecular weight and deacetylation degree determine the viscosity of CS and reduction in molecular weight significantly diminishes CS's viscosity.^41^, ^42^, ^43^ The biocompatibility and biodegradability are other beneficial characteristics of CS.^44^, ^45^, ^46^

The biomedical application of CS nanostructures^47^ has garnered much attention in recent years, especially in cancer therapy, which has been investigated in detail; CS nanoparticles (CS NPs) can mediate drug and nucleic acid therapeutic delivery,^48^ chemotherapy,^49^ phototherapy,^50^ and imaging in cancer treatment.^51^ The redox‐sensitive micelles with carboxymethyl CS decoration can be utilized for NIR imaging of liver cancer cells and simultaneously, photo‐ and chemo‐therapy.^49^, ^52^ Another study evaluated the potential of gold (Au)‐embedded CS nanostructures for delivery of drugs in a pH‐sensitive manner and providing fluorescence imaging.^53^ Notably, the surface modification of CS nanostructures is of importance for nucleic acid therapeutic delivery and synthesizing vectors that can form stable complexes with genetic tools. In a recent study, CS‐Au nanostructures have been applied in photoacoustic imaging‐guided nucleic acid therapeutic delivery and photothermal therapy to exert a synergistic impact for cancer suppression.^54^ The CS can be utilized for the development of pulmonary drug delivery systems. Notably, CS‐based nano‐scale delivery systems for pulmonary delivery are of importance in treatment of related cancers such as lung tumor. Furthermore, it can be used in treatment of infectious diseases such as COVID‐19 that is inflicting nowadays.^55^, ^56^, ^57^, ^58^, ^59^, ^60^ The CS‐based nanostructures have demonstrated great efficacy in prolonged delivery of drugs^61^, ^62^ that is of utmost importance in cancer therapy. Due to CS's positive charge, it can easily form complexes with negatively charged nucleic acids. A recent study has exploited CS‐hyaluronate‐SPION nanoparticles for the delivery of siRNA and EP4 antagonist in cancer therapy; these nanoparticles effectively suppress HIF‐1α/EP4 axis in impairing growth and invasion of tumor cells.^63^ Furthermore, CS NPs can mediate pulmonary delivery of CRISPR/Cas9 system, as a new emerging genetic tool.^56^ Besides cancer therapy, CS has a therapeutic impact in alleviating osteoarthritis^64^ and, recently, a hydrogel system based on lactate‐modified CS has been prepared for osteoarthritis treatment due to its antioxidant activity, where it exhibits high biocompatibility.^65^ Furthermore, CS‐based complexes can ameliorate osteoarthritis by enhancing proliferation rate of chondrocytes and preventing apoptosis.^66^, ^67^ In another study, CS NPs were used for the delivery of rosuvastatin to decrease cholesterol levels upon atherosclerosis treatment and to prevent the development of cardiovascular diseases.^68^ Further, CS NPs can be utilized in anti‐inflammatory formulations by delivery of drugs such as diclofenac sodium^69^ and dexamethasone,^70^ among others. Overall, studies highlight the fact that CS‐based NPs demonstrate good biomedical applications.^71^, ^72^, ^73^, ^74^, ^75^, ^76^ The following sections explore the role of CS NPs in the delivery of anticancer drug—doxorubicin (DOX).

## 3 DOXORUBICIN: MECHANISM OF ACTION AND RESISTANCE

The DOX is an anthracycline antibiotic derived from *Streptomyces peucetius caesius* with high antitumor activity^77^, ^78^, ^79^ as it displays efficacy even at low doses in suppressing different neoplasms.^80^ The animal experiments evaluating anticancer activity of DOX have affirmed its potential in minimizing tumor progression and improving survival of animal models.^81^ Antisuppressive activity of DOX has been successfully demonstrated in preclinical models and clinical trials on various cancers, including leukemia, lymphoma, sarcoma, and urogenital cancers, among others.^80^, ^82^ DOX mainly targets genetic components in nucleus and mitochondria inhibiting the cell growth and division. However, DOX alone is not cell‐selective and affects also the function of healthy cells. This antitumor agent is capable of intercalating with DNA to prevent DNA replication and protein synthesis. Furthermore, DOX stimulates DNA damage in tumor cells by preventing the activity of topoisomerase II enzymes. Additional investigations revealed that DOX enhances the production of reactive oxygen species (ROS) to induce DNA damage and destroy cell membrane via direct interaction.^83^, ^84^, ^85^, ^86^

However, DOX resistance evolution in tumor cells is considered a major challenge as various underlying molecular pathways and mechanisms are responsible for the development of DOX resistance.^87^, ^88^, ^89^ The breast tumor is a heterogeneous cancer with different subtypes and its incidence rate is various based on geographical differences. Although chemotherapy is used for breast cancer treatment, its therapy is still a challenge.^90^ The lncRNA H19 is involved in triggering DOX resistance in breast tumor (in vitro and in vivo) via PARP1 downregulation.^91^ The lncRNA TUG1 decreases miRNA‐9 expression via sponging to induce DOX resistance in breast cancer.^92^ The circRNA‐0002060 mediates the DOX resistance in osteosarcoma via miRNA‐198 downregulation and subsequent increase in the expression level of ABCB1.^93^ The TCF4 and EIF5A2 are other molecular pathways that are affected in cancer cells to regulate DOX chemotherapy response.^93^, ^94^ However, antitumor agents, such as trabectedin and resveratrol among others, have shown potential in reversing DOX resistance.^95^, ^96^

Different studies provide novel insights and pathways for development of DOX resistance. The DOX resistance in osteosarcoma can be mediated by TCF4 overexpression. In this case, circ‐0001721 promotes TCF4 expression via miRNA‐758 downregulation to induce DOX resistance.^94^ Another experiment reveals that circATXN7 increases HOXA11 expression via miRNA‐149‐5p downregulation to increase breast cancer progression and to inhibit DOX resistance.^97^ Furthermore, EMT is responsible for cancer metastasis^98^ and its induction can lead to DOX resistance in tumor cells. Therefore, targeting these pathways can effectively suppress DOX resistance in cancer. For instance, silencing RNF8,^99^ CIP2A,^100^ and miRNA‐21^101^ can inhibit DOX resistance in various tumors and impairs their proliferation. A more advanced strategy in reversing DOX resistance is the application of nanoparticles for targeted delivery of DOX at the tumor site and co‐delivery of DOX with other antitumor agents or nucleic acid therapeutics.^88^, ^102^, ^103^, ^104^

## 4 NANOSTRUCTURES OF CHITOSAN‐DOXORUBICIN

### 4.1 Stimuli‐responsive nanocarriers

#### 4.1.1 pH‐responsive

**FIGURE 2:**
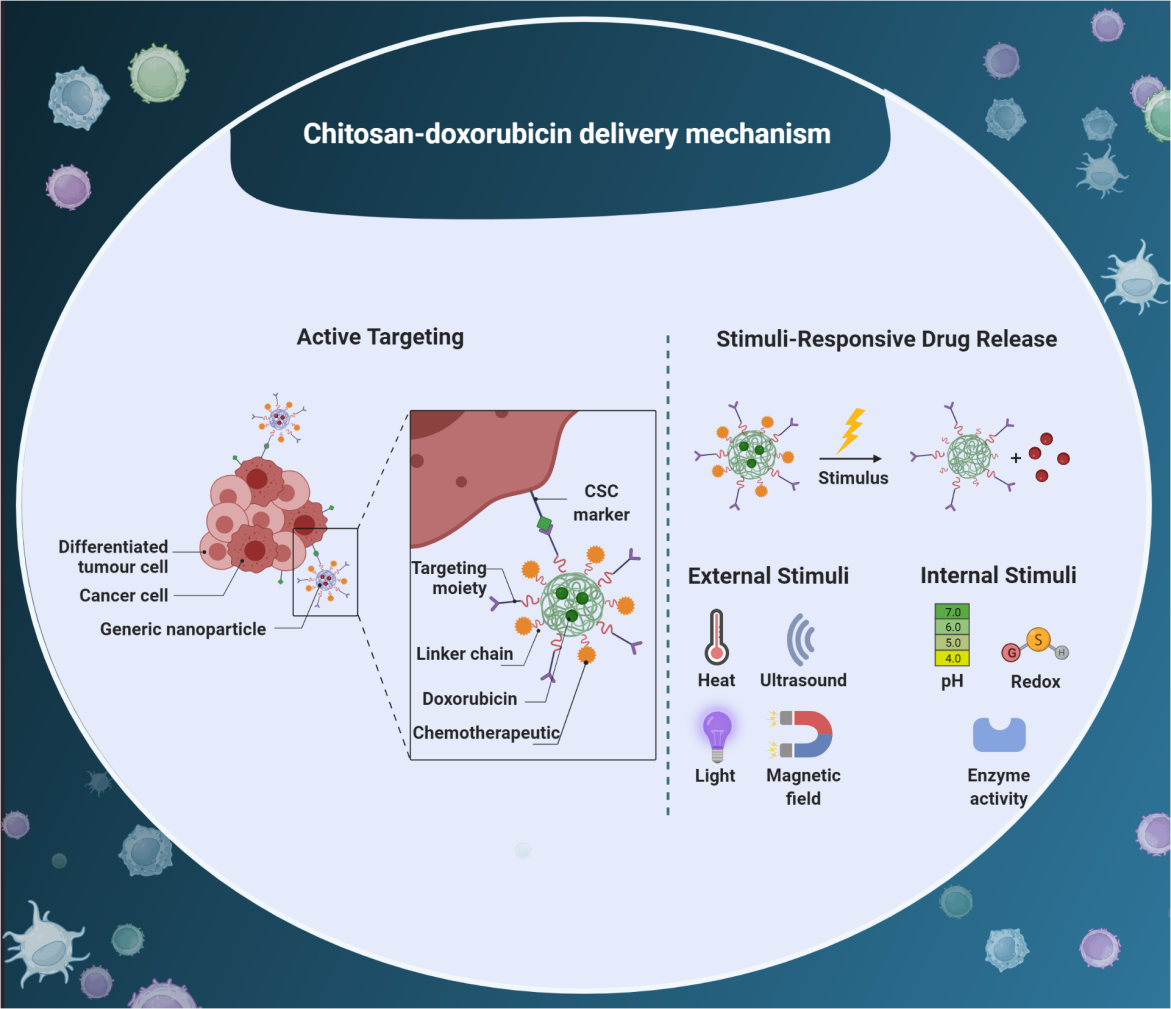
Active targeting and stimuli‐responsive drug release of CS‐doxorubicin nanocarrier. The surface modification of CS‐based nanostructures with ligands can increase their internalization in tumor cells. Besides, External and internal stimuli can be utilized for developing smart nanocarriers in cancer therapy. CSC, cancer stem cell

The tumor microenvironment has a mild acidic pH (approximately 6.5) that is lower than physiological condition and such difference in pH level can be exploited for drug release at tumor site; functionalization of nanomaterials and their drug conjugates can lead to release at this mild acidic pH. Based on this system, the bond between the material and the drug degrades under acidic pH of tumor site to mediate drug release.^105^ Various kinds of bonds including imine, hydrazone, oxime, amide, acetals, and orthoester can be deployed for synthesizing pH‐sensitive nanocarriers and drug release at tumor site^106^ as has been discussed in this section. Figure 2 provides a schematic representation of stimuli‐responsive CS‐based nano‐scale delivery systems in cancer therapy.

Recently, a CS‐based polymeric prodrug, that is pH sensitive and can provide a platform for co‐delivery of DOX and siRNA, has been synthesized. The DOX and Bcl‐2‐siRNA can be conjugated to CS‐modified polymeric nanoparticles and then, synergistic cancer therapy is provided. These nanostructures are internalized by hepatoma cells via glycyrrhetinic acid receptor‐mediated endocytosis. After 10 h, nanostructures can release siRNA and DOX as much as 90.2% and 81.3%, respectively. This CS‐based polymeric prodrug efficiently suppresses tumor progression (HepG2 cells) by 88% via mediating both chemo‐ and nucleic acid‐therapy.^107^

The CS is a pH‐sensitive agent due to the presence of amine groups (—NH_2_) that undergoes protonation in acidic pH^108^, ^109^; higher pH significantly decreases the solubility of CS.^109^ On the other hand, polyvinylpyrrolidone (PVP) is often utilized for the synthesis of nanoparticles, but it significantly decreases the initial burst release.^110^ For overcoming such issues, the combination of PVP and CS has been suggested to improve the solubility of CS at high pH levels and mechanical characteristic of PVP, simultaneously.^109^, ^110^, ^111^ A recent study demonstrated that CS/PVP/α‐Fe_2_O_3_ nanocomposites for the delivery of DOX, where nanoparticles had a spherical structure, and they could load Fe_2_O_3_ in CS/PVP. The nanostructures showed a particle size of 247 nm and due to conjugation of α‐Fe_2_O_3_, they demonstrated prolonged release and increased retention of DOX at tumor site. The CS/PVP‐based nanocomposites release DOX in a pH‐sensitive manner, mimicking pH level of tumor microenvironment and they induced apoptosis to significantly decrease the viability of breast cancer (MCF‐7 cells).^112^

**FIGURE 3:**
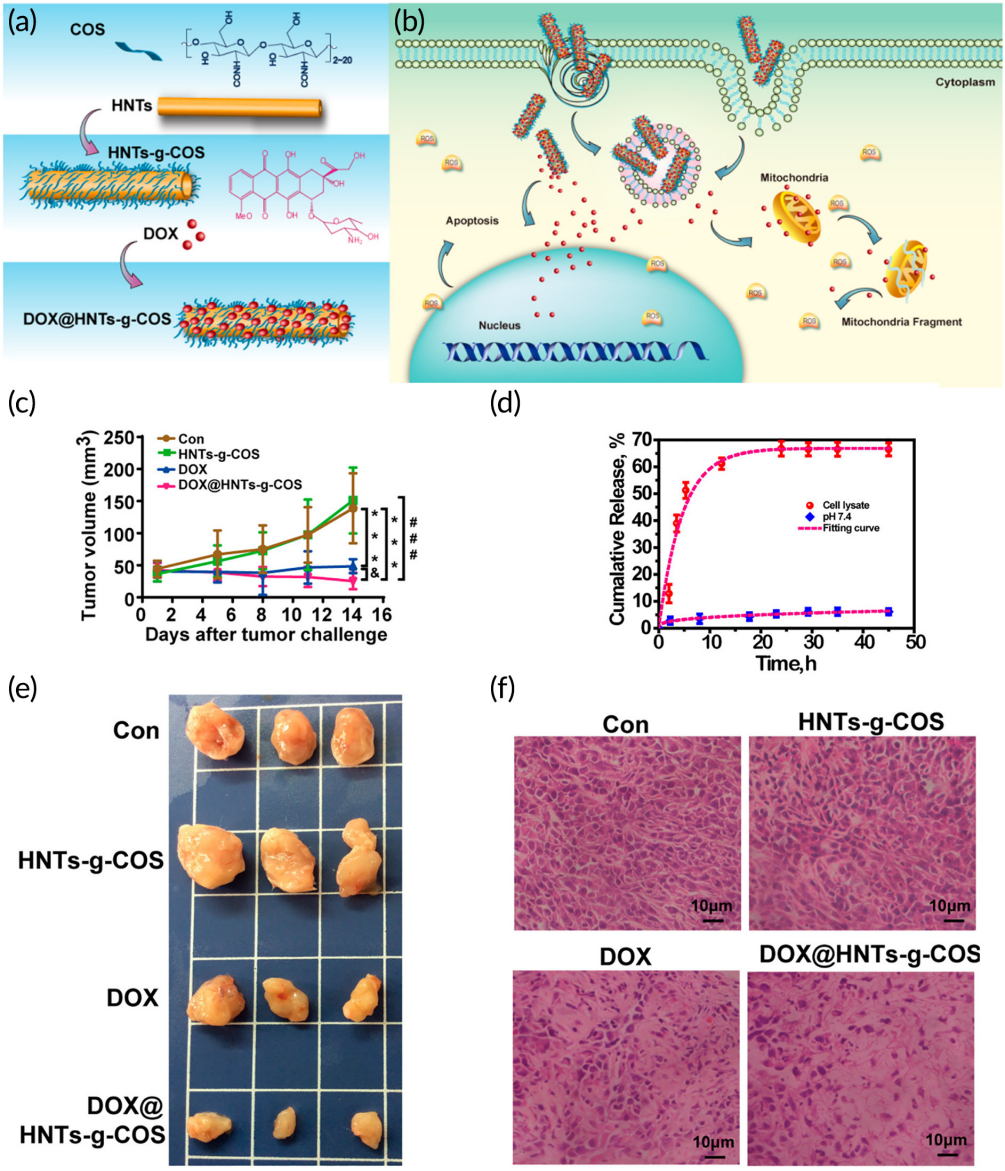
A pH‐responsive CS carrier in combination with halloysite nanotubes for breast cancer therapy. (a,b) The synthesis procedure followed by loading of DOX molecules on the chitosan (COS)‐halloysite nanotubes (HNTs) and the uptake process by which the drug‐loaded carrier induce cytotoxicity toward cancerous cells. (c) Tumor volume changes after being treated with different samples including control (CON), free DOX, unloaded carrier, and DOX‐loaded carrier; significant difference with the control group ***P < 0.001, unloaded carrier at ###*P* < 0.001, and DOX at &*P* < 0.01. (d) The drug release from the carrier at different pH. (e) The excised tumors removed at the end of treatment after being treated with different samples. (f) The histology that was accomplished through H&E staining on the 14th day of treatment. *Source*: Reprinted from Ref 120 with permission from ACS

There are several reasons for using pH‐sensitive nanocarriers for cancer therapy and also delivery of chemotherapeutic agents. The nonspecificity of systemic chemotherapy can negatively affect normal and healthy cells after intravenous injection.^113^, ^114^ Besides, repeating injections can lead to pain, infection, and hospitalization. Therefore, the application of nanoparticles for sustained delivery of chemotherapeutic agents eliminates the need for repeated injections and can improve the chance in the fight against cancer.^114^, ^115^, ^116^ Due to glycolysis phenomenon in the tumor microenvironment and conversion of glucose to lactate, pH level is significantly diminished, which is beneficial for cancer progression.^117^, ^118^ A recent experiment has advanced graphene‐CS nanocomposites for DOX delivery, which were stabilized with bovine serum albumin (BSA); the presence of BSA is beneficial in preventing burst release of drug from CS nanocomposites. They show uniform release over 24 h and can release drug for up to 28 days (84% of drug). Such prolonged release of DOX from CS‐decorated nanocomposites that is pH‐sensitive can improve cytotoxicity against cancer cells.^119^ In another study, a composite structure consists of halloysite nanotubes as a natural aluminosilicate ceramic and CS was designed for pH‐responsive release of DOX for breast cancer therapy. The drug‐loaded carrier showed a sustained release in cell lysate and the mechanism of action was to penetrate into mitochondria followed by inducing damage. Moreover, the IC_50_ of nanocarrier against MCF‐7 cells was found 1.17 μg ml^−1^ lower than that of free DOX (2.43 μg ml^−1^). The in vivo studies revealed that the tumor inhibition ratio of the nanocarrier was 83.5%, whereas the free DOX showed 46.1%. Notably, the treated mice with the DOX‐loaded carrier survived over 60 days without a significant systemic cytotoxicity (Figure 3).^120^

The UV‐triggered injectable CS hydrogels are extensively applied in biomedicine.^121^, ^122^, ^123^, ^124^ Different UV‐crosslinkable CS derivatives have been designed via covalent attachment of UV‐responsive components^125^, ^126^, ^127^, ^128^ to improve their solubility. The “thiol‐ene” click chemistry can be utilized for the development of pH‐responsive UV crosslinkable CS hydrogel. The UV crosslinking ability and pH‐sensitive capacity of CS ensue from allyl groups on C_6_ site and amine groups on C_2_ site. At various pH levels, CS‐based hydrogels show different behaviors, and their swelling and shrinkage can lead to the release of DOX in a pH‐sensitive manner.^129^ The purpose of using CS in the modification of nanoparticles is its capacity in functionalizing or loading various antitumor drugs (DOX), targeting ligands (aptamer), coating polymers and imaging probes.^130^ For this purpose, Au nanoparticles were modified with CS and DNA aptamer to mediate selective targeting to glioblastoma cells. Obtained nanostructures were utilized for delivery of 5‐flourouracil (5‐FU) and DOX in glioblastoma suppression. The prepared CS‐Au NPs had a particle size of 196.2 nm with zeta ζ‐potential of 16.26 mV and they significantly enhanced the cytotoxicity of DOX and 5‐FU against glioblastoma cells and induced cell death and G0/G1 cell cycle arrest.^131^ Based on these results, it can be concluded that CS is a promising agent for the synthesis of pH‐responsive nanocarriers for DOX delivery and cancer suppression^132^ and can be easily functionalized by other ligands to improve the selectivity of NPs toward cancer cells, while simultaneously providing co‐delivery of DOX and other antitumor agents such as 5‐FU. By providing pH‐sensitive feature, these nanocarriers release DOX at the tumor site and also mediate sustained delivery, which are both beneficial in cancer suppression.

#### 4.1.2 Redox responsive

The presence of redox imbalance is another unique feature of tumor microenvironment that is responsible for enhancing tumor proliferation rate. The production of ROS, initiated by inflammatory cells, endothelial cells, and cancer‐associated fibroblasts, induces an aerobic glycolysis and significantly increases tumor progression. On the other hand, glutathione (GSH) is an enzyme that regulates oxidative stress and diminishes ROS levels. In the pharmaceutical industry, there have been efforts in synthesizing redox‐sensitive nanomaterials. Preparation of such nanostructures usually involves incorporation of disulfide as it undergoes degradation by GSH.^133^, ^134^ This section focuses on redox‐sensitive CS‐NPs and disulfide bond decomposition by GSH at the tumor site for DOX release.

As has been discussed in the previous sections, a combination of CS with other agents is applied to synthesize NPs and improve features that are important for cargo delivery. The CS oligosaccharide (CSO) and stearic acid (SA) can be utilized for the preparation of glycolipid‐like copolymer^135^ and ensued CSO‐SA copolymer has high stability that can be further exploited for drug delivery applications^136^, ^137^, ^138^, ^139^ as it exhibits high internalization and cellular uptake.^136^ However, micelles prepared from CSO‐SA have a major challenge to efficiently release the drug in vitro due to slow degradation kinetics of amide bond. To improve drug release profile from CSO‐SA‐based NPs, DOX can be conjugated with CSO‐SA via disulfide bond. This approach is advantageous in synthesizing CSO‐SA‐based nanoparticles that are redox sensitive and due to higher GSH levels in cells, they release DOX to suppress breast cancer progression (MCF‐7 cells).^140^ In another study, carboxymethyl CS (CMC)‐based micelles for delivery of DOX in cancer therapy were prepared. The poly‐*ε*‐caprolactone (PCL)‐SS‐CMC self‐assembled into micelles for improvement of their selectivity against cancer cells (liver and cervical cancers) and were modified with glycyrrhetinic acid (GA). Then, DOX and another antitumor agent known as pheophorbide A (PHA) were loaded on these NPs, which showed a release profile up to 86.3% and 92.1% of DOX and PHA, respectively, after 48 h. This approach is beneficial in enhancing intracellular accumulation of DOX and PHA by providing GA receptor‐mediated endocytosis and redox‐sensitive system.^49^ Although a few experiments have evaluated the potential of redox‐sensitive CS‐based NPs for delivery of DOX,^140^ they highlight the fact that disulfide bond between CS and DOX can be easily degraded in the presence of GSH. This system is biocompatible and its modification with ligands can be performed to promote its potential in DOX delivery.

#### 4.1.3 Light responsive

**FIGURE 4:**
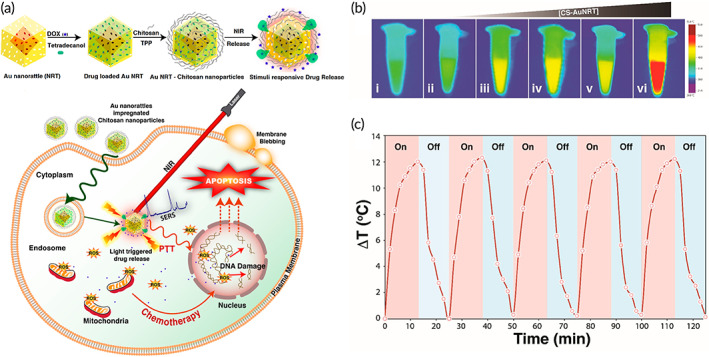
The light‐responsive DOX‐loaded gold‐chitosan nanocomposite. (a) The preparation of CS‐gold nanorattles (AuNRT), DOX loading and the effect of NIR irradiation on the payload release. (b) The thermal images related to the nanocomposite after being irradiated with 785 nm laser for 15 min with various concentrations including (i) water as the control, (ii) 75, (iii) 150, (iv) 200, (v) 500, and (vi) 800 μg ml^−1^. (c) Measurements of temperature increase after applying on/off cycles at a power density of 5 W/cm^2^. *Source*: Reprinted from Ref 143 with permission from ACS

A few experiments have exploited the role of CS‐based nanostructures for light‐mediated release of DOX, which deployed nanobubbles.^141^ The nanobubbles have spherical core–shell structure and their surface can be conjugated with functional groups. The nanobubbles possess enhanced permeability and retention (EPR) effect that is of importance for crossing over endothelial barrier. The nanobubbles can be administered via intravenous route and in order to improve their biocompatibility and biodegradability, surface modification of nanoparticles with PLGA and PCL can be performed.^142^ In an experiment, modification of nanobubbles with CS has been conducted. The CS nanobubbles can release DOX in vitro upon irradiation and significantly enhance uptake of DOX in mammalian cells. These biocompatible nanobubbles can be used for inhibiting breast tumor suppression (MCF‐7) via light‐mediated DOX delivery.^141^ A light‐responsive theranostic platform composed of DOX‐loaded gold nanoparticles and CS was reported for breast cancer therapy. Applying NIR irradiation not only facilitated the payload release in the cancer cells but also caused the gold nanoparticles to elevate the inner temperature up to level, which leads apoptosis. Besides photothermal therapy, it was observed that the liberated DOX caused an oxidative stress through generation of ROS.^143^ Through Figure 4, the preparation and the mechanism of action of the light‐responsive platform are indicated.

#### 4.1.4 Thermosensitive

The thermosensitive hydrogels have garnered much attention in recent years as an implantable delivery system. Thermosensitive hydrogels are injected into tumor site in a liquid state and undergo conversion into a solid gel at body temperature. Besides, hydrogels can provide the prolonged release of antitumor drugs at the tumor site.^144^, ^145^, ^146^ A study describes the synthesis of thermosensitive CS hydrogel to encapsulate liposomal DOX with a particle size of 94.2 nm and encapsulation efficiency as much as 98%. This thermosensitive hydrogel changes to a solid gel at body temperature and provides sustained release of DOX. These hydrogels demonstrated a high safety profile and biocompatibility, and simultaneously, they showed capacity in suppressing tumor progression (H22 hepatoma cells and tumor‐bearing mice).^147^ The application of CS prevents the burst release of DOX from hydrogels. Notably, CS‐DOX conjugate in hydrogels does not affect the antitumor activity of DOX and it is comparable to free DOX. Both in vitro and in vivo experiments have shown the potential of hydrogels containing CS‐DOX conjugate for cancer suppression (A549 lung cancer cells and nude mice).^148^ Furthermore, loading liposomal DOX in hydrogel does not negatively affect the entrapment efficiency of liposomes. For instance, an experiment synthesized CS‐based thermosensitive hydrogel containing liposomal DOX for topical cancer treatment (hepatoma). The entrapment efficiency was 90% and after loading liposomal DOX in the hydrogel, its entrapment efficiency did not change.^149^ Notably, carbon nanotube (CNT)‐CS can be loaded in thermosensitive hydrogels for controlling DOX release. Their exposure to irradiation provides a photothermal effect of CNTs that is beneficial in destroying hydrogel structure and mediating DOX delivery.^150^ Therefore, a thermosensitive hydrogel can be synthesized first for conversion to solid gel at body temperature and in the next step, CS‐carbon nanotubes are loaded into the hydrogel for regulating DOX release upon irradiation.^150^ The succeeding section focuses on multisensitive CS‐based nanocarriers for DOX delivery.

#### 4.1.5 Multiresponsive

**FIGURE 5:**
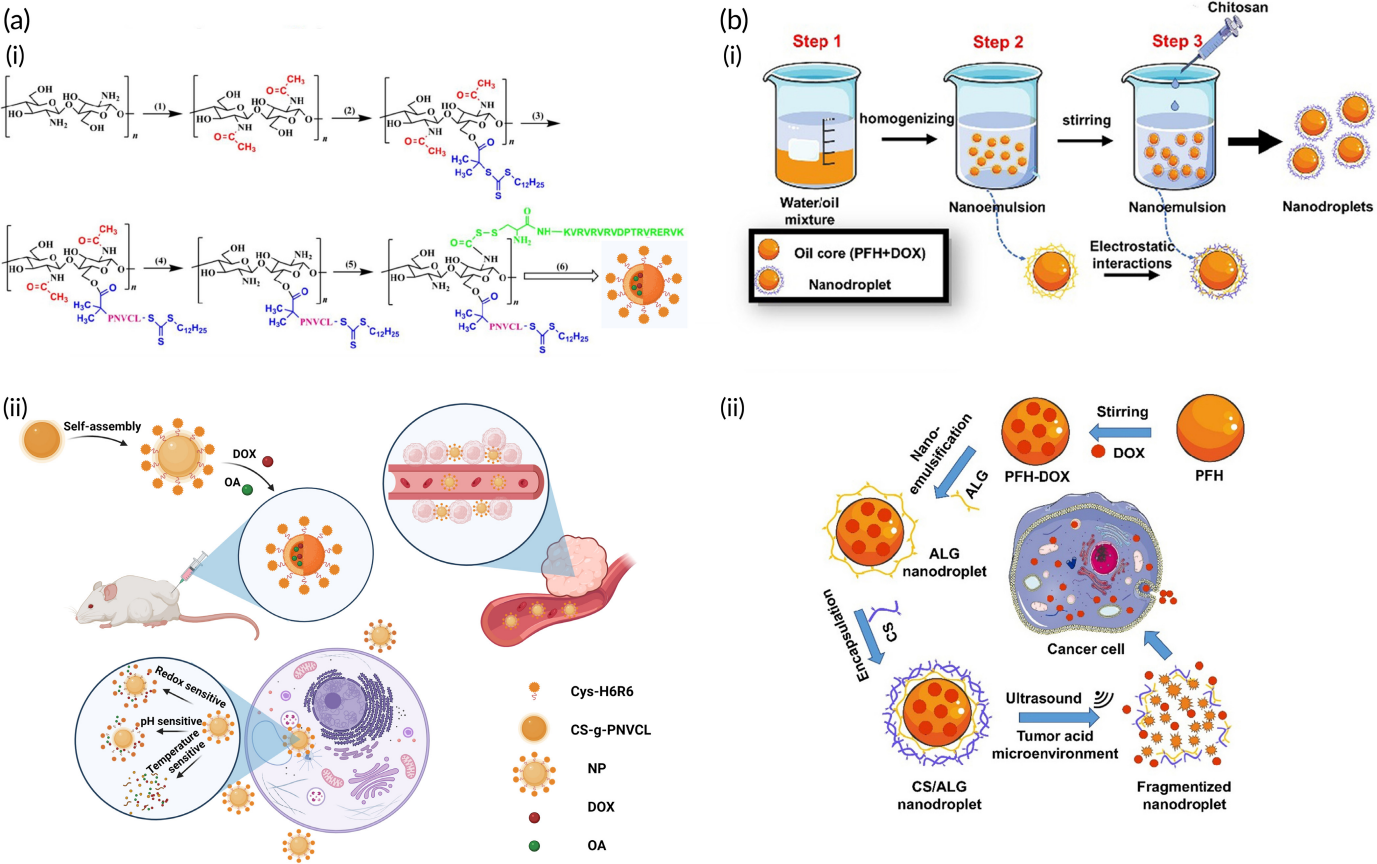
Multiresponsive CS‐NPs for DOX delivery. (a) (i) Step‐by‐step synthesis of multiresponsive (pH, thermo, and redox responsive) DOX/(oleanolic acid [OA[)@functionalized cell‐penetrating peptide (H6R6)‐chitosan (CS)‐g‐poly(*N*‐vinylcaprolactam) (PNVCL) NPs. (ii) The applicability of nanoparticles for anticancer therapy; the improved permeation and retention leads the NPs to remain in the tumor environment followed by triggering triple sensitivity to release doxorubicin (DOX) and OA. *Source*: Reprinted from Ref 151 with permission from Elsevier. (b) (i) Synthesis of DOX‐loaded alginate (ALG)/chitosan (CS) stabilized perfluorohexane (PFH) NPs through nano‐emulsion technique. (ii) Double sensitivity (ultrasound and pH) of the DOX‐loaded nanodroplets against cancer cells. *Source*: Reprinted from Ref 153 with permission from Elsevier

There have been many efforts in developing multifunctional CS‐based NPs for DOX delivery in cancer treatment including nanocomposites that are triple sensitive. For preparing such systems, CS is utilized as a pH‐sensitive agent, g‐poly(*N*‐vinylcaprolactam) (PNVCL) as a thermosensitive agent and H6R6 as a cell‐penetrating peptide. Then, DOX and oleanolic acid (OA) are loaded on nanocomposites with a particle size of 190 nm, the loading efficiency being 13.2% and 7.3% for DOX and OA, respectively. These nanocomposites are accumulated in the tumor microenvironment and the drug is released at the tumor site. The in vitro and in vivo experiments demonstrated the potential of DOX and OA‐loaded CS/PNVCL/H6R6 nanocomposites in suppressing cancer progression and apoptosis induction (SKOV3 ovarian cancer cells and nude mice).^151^ Another strategy for improving the physicochemical properties of CS exploited its conjugation with PEG, which significantly enhances solubility and biocompatibility. The hollow mesoporous silica NPs have been modified with CS and PEG with loading efficiency of 32.8%. The DOX is loaded in PEF‐CS‐silica nanoparticles and there is no release of DOX at low levels of GSH and pH 7.4. However, a mild acidic pH or higher levels of GSH can induce DOX release in breast cancer suppression.^152^ Another study prepared alginate/CS‐based NPs for delivery of DOX that are pH‐ and light‐responsive where alginate improved stability of CS‐based NPs and could release DOX in pH‐ and light‐sensitive manner after irradiation and in mildly acidic pH (Figure 5).^153^ Based on these studies, CS and its combination with other agents can be beneficial in the development of multifunctional nanostructures for DOX delivery **(**Table 1).^159^, ^160^, ^161^

**TABLE 1:** The application of stimuli‐responsive CS‐based nanocarriers for DOX delivery.

Nanovehicle	Stimulus response	Particle size (nm); zeta potential (mV); encapsulation or loading efficiency (%)	Antitumor agent	Remarks	References
Polymeric nanoparticles	pH‐ and thermo‐sensitive	190 nm; 13.2% for DOX and 7.3% for oleanolic acid	Doxorubicin; oleanolic acid	CS provides pH‐sensitive feature and PNVCL leads to thermosensitive feature Accumulation at the tumor site and suppressing ovarian cancer progression Synergistic impact between antitumor compounds	151
PEG‐ and CS‐conjugated hollow mesoporous silica nanoparticles	Redox‐ and pH‐sensitive	230 nm; −11.5 mV	Doxorubicin	Addition of 10 mM of GSH induces DOX release from nanostructures DOX release at mild acidic pH High stability and biocompatibility	152
Alginate/CS‐stabilized nanoparticles	Ultrasound‐ and pH‐responsive	73.3–132.7 nm; 6.41 mV (Step 1), −62.13 mV (Step 2) an d −26.83 mV (Step 3)	Doxorubicin	High biocompatibility Decreasing survival of tumor cells, showing their cytotoxicity	153
GA‐functionalized CS‐based micelles	Redox sensitive	122.4 nm	Doxorubicin; pheophorbide A	Increased cellular uptake by HepG2 cells due to GA modification Providing both photo‐ and chemo‐therapy in cancer suppression	49
CS‐modified Fe_3_O_4_/rGO nanocomposites	pH‐sensitive	‐	Doxorubicin	Promising drug delivery systems for DOX and their modification with folic acid remarkably elevates the accumulation of DOX in cancer cells	154
Aptamer‐functionalized CS‐gold nanoparticles	pH sensitive	196.2 nm; 16.26 mV; up to 96%	Doxorubicin; 5‐fluorouracil	Inducing cell cycle arrest at G0/G1 phase Cell death induction Impairing progression of glioblastoma pH‐sensitive release of DOX and 5‐FU in synergistic tumor ablation	131
CS‐mesoporous silica nanoparticle	pH‐sensitive	155 nm;−1.66 mV	Doxorubicin	CS functions as a gatekeeper and covers surface of MSN DOX release at the tumor site Suppressing cancer progression in vitro and in vivo Apoptosis induction and decreasing proliferation rate	155
Nanomicelle	pH‐sensitive	133.52 nm; 13.5 mV; more than 80%	Doxorubicin; quercetin	Providing lysosomal escape by CS‐based micelles DOX and quercetin release at the cytoplasm of breast cancer cells	156
CS‐tripolyphosphate nanostructures	pH‐sensitive	‐	Doxorubicin	Apoptosis induction Cell cycle arrest at G2/M or S phase Increasing DOX uptake by cervical cancer cells	157
CS‐poly (*N*‐isopropylacrylamide)‐coated mesoporous silica nanoparticles	pH‐ and thermo‐sensitive	70 nm	Doxorubicin	High biocompatibility and stability Suppressing progression of HeLa cells	158
CS nanobubbles	Ultrasound‐responsive	641 nm; +67.12 mV; 54.18%	Doxorubicin	Ultrasound induces the release of DOX from CS‐modified nanobubbles to suppress breast cancer progression	141

Abbreviations: CS, chitosan; DOX, doxorubicin; 5‐FU, 5‐fluorouracil; GSH, glutathione; GA, glycyrrhetinic acid; GO, graphene oxide; MSN, mesoporous silica nanoparticle; PNVCL, poly(*N*‐vinylcaprolactam).

**FIGURE 6:**
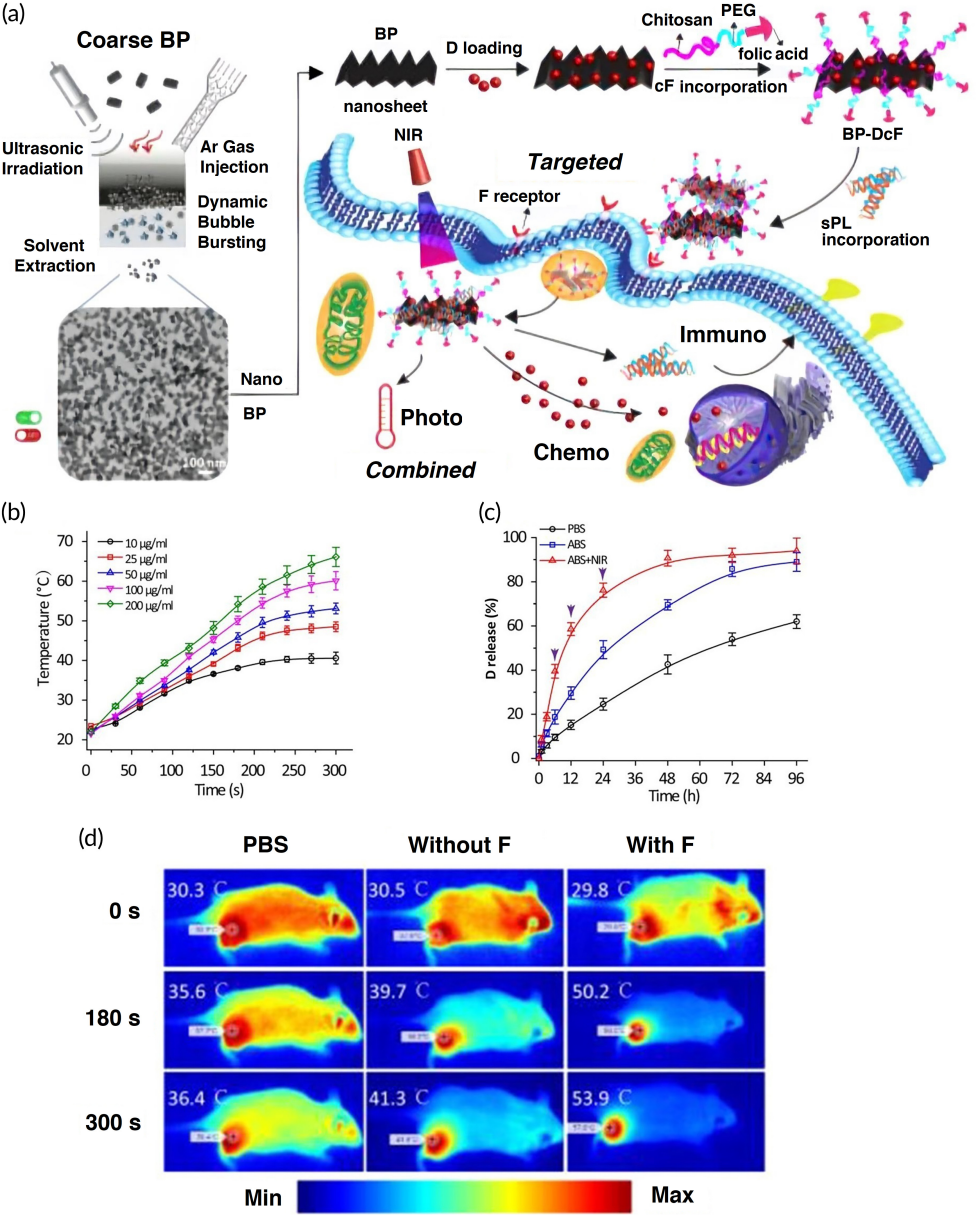
Combination of chemo and photothermal therapies for cancer therapy. (a) An illustration of synthesis of black phosphorus (BP) nanosheets followed by being modified with doxorubicin (D)‐chitosan‐PEG‐folic acid (cF) and small interfering RNA and programmed death ligand 1 (sPL) to form a multifunctional platform called BP‐DcF for cancer therapy. (b) Assessment of temperature increase when the NPs were being exposed to near‐infrared irradiation (808 nm, 1.5 W/cm^2^) for 5 min in a concentration‐dependent manner. (c) The D release profiles from the NPs in different media including acetate‐buffered saline (ABS) pH 5.0 and phosphate‐buffered saline (PBS) pH 7.4. In the case of ABS + NIR, there are some black arrows representing the applied infrared in those time intervals. (d) The photothermal therapy (808 nm, 1.5 W/cm^2^, 5 min) applied in the tumor's sites treated with two samples including without F (Cy5.5‐labeled BP‐Dc@s) and with F (BP‐DcF@s) plus the control group (PBS). *Source*: Reprinted from Ref 164 with permission from ACS

Besides internal‐responsive CS‐NPs for DOX delivery, there is another category termed hyperthermia‐based external‐stimuli drug delivery systems. These systems are responsive to an external stimulus, like magnetic field and light capable of increasing the temperature of the tumor microenvironment followed by killing the cancerous cells.^162^ Moreover, after being triggered by the mentioned stimuli agents, the anticancer drug release rate undergoes a significant increase thus improving the efficiency of the delivery system.^163^ This strategy has been exemplified by a multifunctional chemo‐phototherapeutic delivery system where black phosphorus nanosheets have been adopted as an inorganic light‐responsive material; the DOX has been first loaded onto the nanosheets followed by being surface modified with CS‐PEG and folic acid to form the final platform (BP‐DcF). Next, small interfering RNA and programmed death‐ligand 1 (PL) are encapsulated into the delivery system. Upon irradiation of near‐infrared, a hyperthermia effect and burst release of DOX could be observed and the combination of both chemo and phototherapies culminated in an effective tumor cells apoptosis (Figure 6).^164^

Overall, some technical conclusions about CS‐based nanocarriers for DOX delivery can be provided. Due to protonation of amine groups of CS at acidic pH that is similar to tumor microenvironment pH, the pH‐sensitive release of DOX occurs upon CS modification of nanocarriers. Notably, by loading some kinds of nanoparticles such as Fe_3_O_4_ in CS‐based nanocarriers, multifunctional nanoarchitectures are designed that can provide stimulus‐responsive release of DOX and simultaneous imaging.

### 4.2 Reversing drug resistance

There are several reasons responsible for role of NPs in reversing chemoresistance. Frequent application and high doses of chemotherapeutic agents can result in drug resistance development and in targeted delivery, a low amount of anticancer agent is loaded that reduces chance of drug resistance, while it maintains tumor‐suppressor activity.^165^, ^166^, ^167^ Additionally, increasing accumulation of chemotherapeutic agents in tumor cells prevents drug resistance and NPs are promising in promoting cellular uptake of anticancer agents. There have been some efforts in application of CS‐based nanostructures for suppressing DOX resistance; diverse applied strategies are reviewed in this section.

Preventing drug efflux can be beneficial in reversing drug resistance. One of the factors involved in mediating drug efflux is P‐glycoprotein (P‐gp), as a transmembrane transporter belonging to the family of ATP‐binding cassette (ABC) transporters. The enhanced activity and expression of P‐gp are responsible for inducing the drug resistance.^13^, ^167^ In this case, CS‐*g*‐d‐α‐tocopheryl polyethylene glycol 1000 (TPGS) nanostructures have been developed for DOX delivery. The nanoparticles are synthesized using a modified solvent extraction/evaporation method mixed with ionic cross‐linking. The ensuing nanostructures showed a particle size of 140–180 nm with loading efficiency of 40% for DOX and being pH‐sensitive, they could release DOX at the tumor site in response to mild acidic pH of the tumor microenvironment. The reason for using TPGS is its potential to inhibit P‐gp activity. Therefore, CS‐*g*‐TPGS NPs suppress P‐gp activity and significantly decrease the ATP levels that are beneficial in preventing DOX efflux. Furthermore, they provide an increase in internalization of DOX for apoptosis induction and decrease the survival of tumor cells (liver and breast cancers).^168^ These mechanisms participate in suppressing DOX resistance in cancer cells.

To investigate efficacy of CS NPs in preventing the progression of DOX‐resistant cancer cells, a study developed CS‐dextran sulfate‐coated PLGA‐PVA NPs for DOX delivery and inhibiting the progression of DOX‐resistant breast tumor cells; spherical NPs showed high stability with ζ‐potential of +2.89 mV. The double coating of PLGA‐PVA nanostructures with CS and dextran sulfate is beneficial in inducing cytotoxicity against breast cancer cells (MCF‐7 cells); these ensued NPs significantly enhanced DOX uptake in MCF‐7 cells. Furthermore, they induced apoptosis, DNA damage and S cell cycle arrest, while they reduced invasion of parental cells that are beneficial in suppressing DOX resistance. The upregulation of Bax, PARP1, p21, and p23 has been observed upon using DOX‐loaded CS‐dextran sulfate‐coated PLGA‐PVA nanoparticles.^169^ The deployment of CS promotes the stability of NPs and pH‐sensitive capacity that are both vital for overcoming DOX resistance.^170^ Therefore, CS‐NPs can effectively suppress DOX resistance,^171^, ^172^, ^173^ and based on the studies, apoptosis induction, cell cycle arrest, inhibiting migration, promoting cellular uptake, preventing drug efflux via P‐gp down‐regulation and affecting tumor‐promoting and tumor‐suppressor pathways can be followed.

### 4.3 Doxorubicin and gene delivery

**FIGURE 7:**
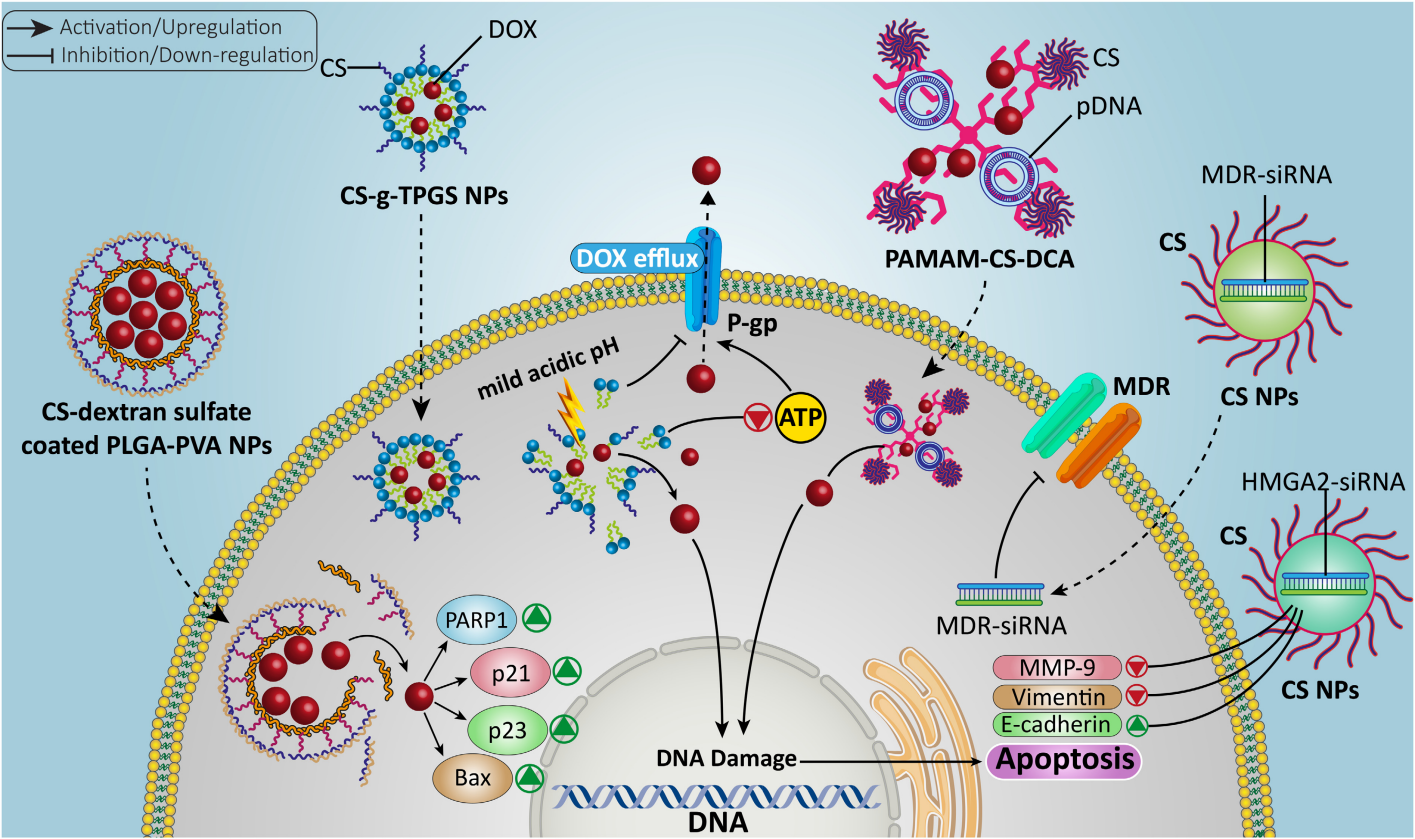
The CS‐based nano‐scale delivery systems for the purpose of gene and DOX delivery in cancer. The nucleic acids lead to apoptosis, DNA damage, and alterations in expression of genes to sensitize tumor cells to chemotherapy. Besides, efflux of chemotherapeutic agents is prevented via reducing activity and expression of P‐gp. Then, CS‐based nano‐scale delivery systems increase accumulation of anticancer drugs in tumor cells and promote their capacity in decreasing tumor progression

Gene therapy is a new emerging field for disease management and it can be considered as an option for treatment of diseases that are incurable with conventional medicines.^174^, ^175^ Various nucleic acid drugs have been developed for purpose of gene therapy and have shown promising results.^176^ However, gene therapy has its own drawbacks and naked genetic tools show limited efficacy in disease treatment, especially cancer. Thus, there is a need for developing nanoscale delivery systems for the delivery of genes. Furthermore, nanoarchitectures can provide co‐delivery of genes and drugs for synergistic disease therapy that is common in cancer treatment.^177^, ^178^, ^179^, ^180^, ^181^ This section focuses on CS‐based NPs for co‐delivery of DOX and genes in cancer suppression (Table 2 and Figure 7).

**TABLE 2:** CS‐based nanocarriers for co‐delivery of DOX and genes in cancer suppression.

Nanovehicle	Gene	Antitumor agent	Particle size (nm); zeta potential (mV); encapsulation efficiency (%)	Remarks	Refernces
PAMAM‐CS‐DCA nanoparticles	pDNA	Doxorubicin	140–220 nm	Encapsulating DOX at core Loading pDNA on PAMAM shell with positive charge Transfection efficiency as much as 74% Exerting synergistic activity and suppressing cancer progression	182
CS‐based nanostructures	IL17RB‐siRNA	Doxorubicin	114 nm; 10.1 mV	Inducing apoptosis in breast cancer cells and suppressing their migration and invasion siRNA promotes cytotoxicity of DOX against tumor cells Targeted delivery by nanocarriers Decreasing expression levels of Bcl‐2 and NF‐κB	183
Micellar polyplexes	MDR‐1‐siRNA	Doxorubicin	92 nm; 7–10 mV	Impairing progression of breast cancer cells (4 T1 cells) Enhancing survival of animal model Down‐regulating MDR1 expression and promoting DOX sensitivity of tumor cells	184
CS nanostructures	HMGA2‐siRNA	Doxorubicin	110–174 nm; 11.6–13.2 mV; up to 78%	Stability against serum and heparin Inducing cell death Decreasing expression level of HMGA2, vimentin and MMP‐9, while promoting E‐cadherin levels in impairing progression of colorectal tumor cells Exerting synergistic impact between DOX and siRNA	185
LDL‐CS nanoparticles	MDR‐1‐siRNA	Doxorubicin	23.4 nm; −1.65 mV; 71.06%	Protecting siRNA against macrophage phagocytosis Decreasing MDR1 expression Accumulating at tumor site Suppressing liver cancer progression	186
CS‐gold nanoparticles	MDR‐1‐siRNA	‐	94.5 nm	Reducing expression level of MDR‐1 and P‐gp to significantly enhance DOX internalization in breast cancer cells	187
CS nanoparticles	IGF‐1‐siRNA	Doxorubicin	176 nm; 11 mV	Apoptosis induction due to synergistic impact between siRNA and DOX and subsequent impairment of A549 progression	188
Glycol CS nanoparticles	Bcl‐2‐siRNA	Doxorubicin	290 nm; 0.86 mV; up to 93%	Release of cargo in a pH‐sensitive manner Reducing cancer cell survival via Bcl‐2 down‐regulation and enhancing DOX sensitivity	189
CS‐based nanostructures	HMGA2‐siRNA	Doxorubicin	207 nm; 16.3 mV	Apoptosis induction Impairing migration and invasion of cancer cells Decreasing HMGA2, vimentin and MMP‐9 levels Enhancing E‐cadherin levels	190
Trimethyl CS nanostructures	HMGA2	Doxorubicin	120–205 nm; 13.5–17.3 mV; up to 80.2%	Impairing migratory ability of breast cancer cells via co‐delivery of siRNA and DOX Suppressing breast tumor proliferation	191

Abbreviations: CS, chitosan; DOX, doxorubicin; DCA, deoxycholic acid; HMGA2, high mobility group A2; MDR1, multidrug resistance 1; MMP, matrix metalloproteinase; NF‐κB; nuclear factor‐kappa B; PAMAM, poly(amidoamine); P‐gp, P‐glycoprotein.

Although CS has a positive charge and can form stable complexes with genes possessing a negative charge, it has a hydrophilic segment that reduces its affinity toward genes and shows poor solubility.^192^ To overcome these drawbacks, a dendronized CS derivative (poly(admidoamine) [PAMAM]‐CS) and PAMAM have been grafted into CS.^193^ A recent study prepared PAMAM‐CS and then embedded hydrophobic deoxycholic acid (DCA) into CS backbone. The final PAMAM‐CS‐DCA has an amphiphilic nature and can self‐assemble into cationic nanostructures. The resulting nanocarriers demonstrated a particle size of 140–220 nm. The DOX as a hydrophobic drug is loaded in the core of NPs, while plasmid DNA (pDNA) forms a stable complex with a positively charged PAMAM shell. These NPs exhibited high transfection efficiency (74%) in 293 T cells and they may be considered promising for delivery applications.^182^ Another important strategy for co‐delivery of DOX and gene is loading CS‐gene conjugate and DOX in other vectors. For this purpose, double‐walled microspheres can be prepared and then, DOX and CS‐DNA nanostructures containing *p53* gene can be loaded to provide simultaneous chemotherapy and gene therapy of tumor cells.^194^, ^195^

The CS has been considered as a promising agent for modification of NPs for the delivery of small interfering RNA (siRNA) due to its positive charge, capacity in generating stable complex, high biocompatibility, and effective suppression of tumor cells.^177^ The interest for the delivery of siRNA is due to circumventing a number of challenges such as poor internalization in tumor cells, prolonging blood circulation and preventing siRNA degradation by RNase enzymes.^13^, ^196^, ^197^, ^198^ The CS‐based nanostructures can provide a platform for the co‐delivery of DOX and siRNA in cancer chemo‐ and gene therapy. A study described the preparation of DOX‐loaded mixed micelles as core via thin‐film hydration method and then used CS as a coating agent and finally, complexation with multidrug resistance (MDR)‐siRNA has been made. To improve the selectivity toward cancer cells, modification of nanostructures with folic acid was accomplished. Due to the presence of CS, these nanoparticles are pH‐sensitive and can release MDR‐siRNA at the tumor site and significantly promote its internalization. Furthermore, the combination of DOX and MDR‐siRNA exerts a synergistic impact and suppresses tumor progression in vitro and in vivo (4 T1 breast tumor cells and Balb/c mice).^184^ Although CS is an ideal agent in the synthesis of NPs, it has also some problems that need to be addressed. The CS nanostructures have high complexity, high degree of mutability, and complicated extraction process.^199^, ^200^ Therefore, carboxymethyl dextran (CMD) can be used to improve the features of CS NPs in drug and nucleic acid therapeutic delivery. An experiment has prepared CMD‐CS nanostructures for colorectal tumor suppression via siRNA and DOX co‐delivery and the nanosystem exhibited high encapsulation efficiency for both high mobility group A2 (HMGA2)‐siRNA (78%) and DOX (75%) and can induce cell death. Furthermore, HMGA2‐siRNA delivery by CMD‐CS nanoparticles suppresses metastasis of colorectal tumor cells via down‐regulating MMP‐9 and vimentin, and upregulating E‐cadherin that are beneficial in enhancing DOX cytotoxicity.^185^

In addition to siRNA, short hairpin RNA (shRNA) has been utilized for regulating gene expression. Overall, shRNA has similar function to siRNA and is capable of reducing the expression level of target gene in cancer therapy.^201^, ^202^, ^203^, ^204^ A study synthesized magnetic mesoporous silica nanoparticles (M‐MSNs) and then conjugated them to DOX and chlorin e6 as a photosensitizer. Subsequently, this nanosystem was conjugated to CS and alginate to provide a pH‐sensitive platform and to improve adsorption of P‐gp‐shRNA. This nanosystem with a particle size of 280 nm can offer simultaneous chemotherapy, gene therapy and phototherapy; the release of drug at the tumor site (pH sensitive) and exposure to irradiation significantly enhances the production of singlet oxygen. The high cellular uptake after intravenous injection in tumor‐bearing mice with the synergistic impact between P‐gp‐shRNA, DOX and phototherapy in breast cancer therapy has been illustrated.^205^

Another nucleic acid therapeutic approach is to affect the expression level of microRNAs (miRNAs), as their aberrant expression mediates initiation, development, and progression of various cancers.^206^, ^207^, ^208^, ^209^, ^210^, ^211^, ^212^ The downregulation of miRNA‐34a is observed in breast tumor and promoting its expression could be beneficial in impairing tumor progression. CS‐based nanocarriers can provide co‐delivery of DOX and miRNA‐34a in breast tumor suppression. This combination therapy significantly reduces the Bcl‐2 expression to induce apoptosis. Furthermore, surface modification of CS nanoparticles by hyaluronic acid selectively targets breast cancer cells overexpressing CD44 receptor.^213^ Therefore, CS nanostructures can be utilized in nucleic acid therapeutic delivery in enhancing cytotoxicity of DOX against cancer cells.^205^, ^214^, ^215^, ^216^ Notably, there has been no instance of the co‐delivery of DOX and CRISPR/Cas9 in cancer chemotherapy and future studies can focus on this aspect.

The co‐delivery of DOX and nucleic acids appears to be promising for purpose of effective cancer chemotherapy. There are some underlying reasons for this combination therapy. There are a number of tumor‐promoting factors in tumor cells such as IL‐17RB, IGF‐1R, MDR1, Bcl‐2, and HMGA2, among others, that can mediate resistance of cancer cells to DOX chemotherapy. Therefore, co‐application of nucleic acids for downregulation of these oncogenic factors can sensitize tumor cells to DOX chemotherapy. The surface modification of nanocarriers with positively charged CS leads to proper interaction with negatively charged nucleic acids and forms stable complexes. Furthermore, CS provides a site for conjugation to ligands to selectively target receptors overexpressed on tumor cells.^183^, ^186^, ^187^, ^188^, ^189^, ^190^ These benefits advocate application of CS‐based nano‐scale delivery systems for DOX chemotherapy.

### 4.4 Doxorubicin and antitumor drug delivery

In addition to gene therapy as a promising strategy in promoting cytotoxicity of DOX against tumor cells, there have been attempts to recognize drugs that can exert synergistic impact with DOX in tumor suppression. The most well‐known mechanism is that a certain antitumor agent induces DNA damage and apoptosis in cancer cells and then, the pathway is paved for DOX to inhibit tumor cell growth and invasion.^217^, ^218^ This section focuses on DOX and drug co‐delivery by CS NPs in cancer therapy.

Lung cancer is a leading cause of death worldwide and based on new estimates, it is the most common cancer in both males and females.^219^ These malignant cells can attain resistance to various chemotherapeutic agents and different underlying pathways and mechanisms are responsible for drug resistance in lung cancer.^8^, ^220^, ^221^, ^222^ A recent experiment has described the preparation of glycol CS NPs for the co‐delivery of DOX and celecoxib (CXB) in lung cancer suppression. For selective targeting of lung cancer cells, DOX‐ and CXB‐loaded CS NPs are modified by hyaluronic acid (HA) to target CD44 receptor; ensued CS NPs have a uniform spherical shape with a particle size of 150 nm. Further, this nanosystem is pH‐sensitive and can release DOX and CXB in response to mild acidic pH of the tumor microenvironment (pH 4–6). This combination therapy exerted synergistic impact and suppressed proliferation, migration, and inflammation in lung cancer.^223^ Another study has synthesized UiO‐66 metal organic framework (MOF) for co‐loading DOX and folic acid and then loaded them in CMD/poly ethylene oxide (PEO)/polyurethane core–shell nanofibers for regulated release of drugs. This nanosystem induced cell death in breast cancer (MCF‐7) cells and significantly impaired cancer progression.^224^

The interesting theme is the potential of plant derived‐natural compounds in reversing the DOX resistance in cancer suppression. The curcumin is derived from *Curcuma longa* and can induce apoptosis in tumor cells and it inhibits migration and proliferation that are beneficial in promoting chemosensitivity of tumor cells.^8^, ^222^, ^225^, ^226^, ^227^ The inhalable bioresponsive CS microspheres have been developed for the co‐delivery of soluble curcumin and DOX in lung cancer therapy. These NPs bear elastin as a stimuli‐responsive agent. At pH 5.5, CS microspheres release DOX due to existence of elastase enzyme. Notably, curcumin is also released from CS microspheres by the function of elastase enzyme and independent of pH level. These nanoparticles can provide co‐delivery of curcumin and DOX that is beneficial in exerting synergistic therapy and inducing apoptosis in A549 cells. Furthermore, this combination and targeted delivery can reduce IC_50_ to 3.4 μM compared to 6.5 μM of NPs lacking elastin.^228^ Another experiment has prepared CS liposomal nanocarriers for co‐delivery of DOX and rapamycin (RAPA). At the first step, a conjugation of glycol CS and DOX is formed and then, electrostatic interaction mediates complexation between glycol CS‐DOX and docosahexaenoic acid RAPA‐liposomes. The resulting nanoparticles had a particle size of 131.3 nm with ζ‐potential of −14.5 mV, loading efficiency being 4.1% and 6.2% for DOX and RAPA, respectively. These glycol CS liposomes are stable and exhibit pH‐sensitive behavior, capable of releasing drugs at the tumor site and exerting synergistic breast cancer therapy.^229^ Based on these experiments, CS NPs can mediate co‐delivery of DOX with other antitumor agents. Although a few studies have been performed, more efforts should be made in co‐delivery of DOX with other well‐known antitumor agents such as cisplatin, docetaxel, paclitaxel, and resveratrol among others. Furthermore, other kinds of nanocarriers such as CS‐based micelles and carbon nanomaterials should be evaluated in terms of co‐delivery of DOX and other drugs.

### 4.5 Lipid nanoparticle modification

Liposomes are synthetic lipid NPs that have been first discovered in the 1960s and comprise a lipid bilayer with an aqueous core.^230^ The liposomal nanocarriers can be exploited for the delivery of both hydrophilic and hydrophobic drugs. The hydrophilic drugs can be loaded in the core, while hydrophobic drugs can be loaded in lipid bilayer. The drugs loaded in liposomes are protected against inactivation in blood circulation, dilution, and degradation. The clinical application of liposomes has some impediments such as their rapid clearance, immune system activation, and accumulation in other organs.^231^ Recent experiments have exploited liposomes for delivery of DOX or its co‐delivery with other agents such as hispolon, curcumin, and linalool in combination cancer therapy.^232^, ^233^, ^234^, ^235^ Therefore, liposomal nanocarriers can provide delivery of DOX at the tumor site and this section focuses on CS‐modified liposomes in DOX delivery.

An experiment has conjugated DOX to amphiphilic stearolylspermine anchor to produce a prodrug. Then, this prodrug has been loaded into liposomal nanocarriers for colorectal cancer therapy. For improving the stability of DOX‐loaded liposomes, their modification with CS and PEG has been made. Furthermore, stearoyl chains promote local microfluidity of liposomes and spermine via amine groups interacts with phosphate groups of lipids in improving liposome stability. In addition to stability, CS‐PEG modification of liposomes prevents aggregation and this coating mediates charge neutralization. These neutral liposomal nanocarriers demonstrated cytotoxicity against A549 (lung cancer) and Caco‐2 cells (colorectal tumor) and they showed high stability.^236^ Another study described the preparation of liposomes using hydrogenated soy phosphatidylcholine (HSPC) and cholesterol (CHOL) and glycol CS conjugation has been performed during film preparation. Then, DOX was embedded in preformed liposomes via transmembrane pH gradient loading strategy. These CS‐based liposomes enhance internalization of DOX in tumor cells (HT1080 cells) and were successful in enhancing the therapeutic efficacy of DOX in vitro and in vivo. This system was pH‐sensitive and could be exploited for the treatment of other cancer types.^237^

Another kind of lipid‐based nanoparticles that can be utilized for cancer therapy are micelles as they are considered as promising vectors in drug delivery and cancer treatment due to their ease of synthesis and chemical modification. Furthermore, the size of micellar NPs is tunable and they can enhance drug solubility in water and significantly promote blood circulation of drugs. The increased bioavailability of the drug, lowering adverse impacts and high accumulation at tumor site are other benefits of using micelles for drug delivery. The micelles can provide pH‐responsive release of DOX at the tumor site and provide co‐delivery of DOX with other antitumor agents such as cisplatin for synergistic cancer therapy. Furthermore, surface modification of micelles, for instance, with phenyboronic acid promotes selectivity toward tumor cells and enhances the tumor‐suppressor activity of DOX.^238^, ^239^, ^240^, ^241^, ^242^

The DOX‐loaded micelles can be prepared using alginate and CS in a water‐in‐oil emulsion method with a spherical particle size of 80 nm. This is an interesting method for loading DOX in nanocarriers and uses an aqueous phase dispersed in a cyclohexane/dodecylamine organic phase. These nanocarriers showed high cellular uptake by breast cancer cells and can suppress proliferation of 4 T1 cells.^243^ The CS‐modified micelles can also provide co‐delivery of DOX and curcumin in liver cancer therapy. The CS‐cystamine‐poly(ε‐caprolactone) copolymer micelles have been prepared for curcumin and DOX co‐delivery and then, modification with GA has been performed in enhancing their cellular uptake. They showed drug loading efficiency of 19.8% and 8.9% for DOX and curcumin, respectively. They had a spherical shape with a particle size of 110 nm. The GA modification enhanced its internalization in cells via endocytosis and exposure to the tumor microenvironment induced changes in charge of nanocarriers from negative to positive. These CS‐modified micelles are pH‐ and redox sensitive and 10 mM of GSH induces the release of DOX (80.6%) and curcumin (67.2%). This combination therapy exerts a synergistic impact and is beneficial in suppressing the progression of hepatoma cells.^244^ Overall, CS derivatives that can self‐assemble into micelles, are able to provide nanocarriers that are biocompatible, have low immunogenicity, and provide nanoplatforms for drug delivery.^245^, ^246^, ^247^ An experiment has prepared *N*‐succinyl‐*N*′‐octyl chitosan (SOC)‐based micelles for DOX delivery and increasing the ocetyl chain amount, promotes capacity of these micelles in DOX loading. Drug loading and ocetyl chain number determine the size of micelles and they have a particle size of 100–200 nm. They showed high antitumor activity against various cancer types including HepG2, A549, BGC, and K562 cells.^248^ Hence, similar to liposomes, micellar nanoparticles are potential vectors for DOX delivery and cancer suppression as well as preventing drug resistance development.^249^, ^250^

### 4.6 Metal nanoparticle modification

Metal–organic frameworks (MOFs) can be considered as ideal options for drug delivery due to their nanoscale size, high surface area, and porosity as well as adjustable size.^251^, ^252^ To improve the property of MOFs in drug release, their modification with polymers has been performed; CS modification of MOFs renders them pH‐sensitive feature and provides a condition for sustained release of DOX in cancer chemotherapy. Furthermore, CS can be functionalized by folic acid (FA) for selective targeting of tumor cells overexpressing folate receptor. Then, DOX can be loaded in CS‐modified MOFs with a high drug loading capacity (1.63 g). Notably, MOFs can encapsulate carbon dots for providing imaging. These CS‐based metal NPs provide simultaneous chemotherapy and bioimaging that are beneficial in cervical cancer treatment.^253^ When exposed to mild acidic pH of the tumor microenvironment, CS layers located on the surface of MOFs would collapse and swell, leading to the release of DOX at the tumor site and subsequent breast cancer suppression.^254^

The interesting note is the modification of Fe_3_O_4_ nanoparticles with CS that provides a drug delivery system that can mediate bioimaging. The surface modification of Fe_3_O_4_ with CS is of importance for loading DOX. The CS can form a stable complex with DOX and produces NH_2_‐Zn(II)‐DOX structure. Exposing to certain pH destroys the bond between DOX and CS and leads to the pH‐sensitive release of DOX and Fe_3_O_4_ nanoparticles can provide magnetic resonance imaging.^255^ As has been mentioned in the Introduction section, CS can undergo changes by chemicals and enzymes. There have been efforts in the chemical modification of CS and improving its properties. It has been reported that CS modification with guanidine moieties significantly elevates its intracellular accumulation; guadinylated CS can form chelates with copper via copper‐nitrogen coordination. The CS‐copper complexes enhance the production of ROS in impairing lung cancer progression and exert synergistic cancer therapy with DOX.^256^ Based on these experiments, modification of metal‐based nanostructures with CS improves their biocompatibility and loading of DOX. Notably, further progress can be made by the modification of CS‐metal NPs. A recent experiment has synthesized CS‐Au nanostructures and for enhancing their selectivity toward lung (A549) and breast (4 T1) cancer cells, their modification with nucleolin aptamer (AS1411) was conducted. These nanocomplexes targeted the tumor cells and effectively penetrated into them, leading to DOX accumulation and subsequent cancer progression suppression.^257^ Therefore, CS is used as a reducing and stabilizing agent for loading DOX on Au NPs and further modification of CS‐Au NPs with polyethylene glycol promoters their blood circulation that is of importance for elevating cytotoxicity of DOX against lung cancer cells.^258^ Taking everything together, efficacy of metal‐based NPs for DOX delivery and cancer suppression can significantly enhance by CS modification.^259^, ^260^, ^261^

### 4.7 Carbon‐based nanoparticle modification

A recent experiment has provided novel insights about the surface modification of graphene composites by CS and its impact on DOX conjugation and release. The concentration or pH of the solution (level of CS protonation) affects the aggregation and dispersion of CS on the surface of graphene composites. At low concentration levels of CS and DOX, the bare surface of graphene composites enhances. Increasing the numbers of —NH_2_ CS and DOX molecules promotes the adsorption of DOX on bare surface areas of graphene and mediates encapsulation of DOX by CS clusters on the surface or results in conjugation with CS chains. On the other hand, when levels of —NH_3_ CS and DOX increase, there will be higher positive charges and lower bare surface area of graphene that can provide conditions for the release of DOX. Therefore, at mild acidic pH of the tumor microenvironment, protonation of CS occurs and leads to the release of DOX from nanocarriers.^262^ Graphene oxide (GO) is a derivative of graphite or graphene and has a two‐dimensional plate‐like structure. The GO sheets have both sides and due to their large surface area, they are considered as promising structures for the delivery of antitumor drugs. Furthermore, aromatic molecules and nucleobases can be loaded on GO composites via π–π stacking and hydrogen bonding interactions.^263^, ^264^ For improving the biodegradability of CS and its solubility, conjugation of CS with other agents such as acrylic acid (AA) and itaconic acid (IA) monomers has been performed to improve its functionality via COOH group. Furthermore, modification by folic acid (FA) selectively targets tumor cells overexpressing folate receptor. A recent experiment has first converted GO sheets to amine‐functionalized GO (AGO) to provide a function of a cationic polyelectrolyte. Next, CS and FA conjugation via *N*, *N*′‐dicyclohexylcarbodiimide was performed. Then, CS was chemically modified by AA and IA monomers by binding to COOH group via ethyleneglycol dimethacrylate as cross‐linker and potassium peroxydisulfate as an initiator. Subsequently, DOX was embedded into FA‐chemically modified chitosan (CMCS)/AGO nanocomposites via π–π stacking interactions. The prepared nanocomposites demonstrated drug loading capacity as much as 95% and they released DOX at a pH‐sensitive manner (release of DOX at pH 5.3 compared to pH 7.4). These DOX‐loaded nanoparticles were able to significantly reduce the viability of tumor cells including HeLa and MCF‐7 cells.^265^

Another experiment has prepared injectable hydrogels for the controlled release of DOX. This hydrogel is based on cross‐linking of graphene, CS, and cellulose nanowhiskers where Schiff base reaction by a synthetic dialdehyde has been used. This hydrogel is responsive to pH and other stimuli by adding benzaldehyde and amino acid cysteine and can be administered subcutaneously to deliver both DOX and curcumin in synergistic cancer therapy.^266^ In addition, CNTs can be modified with CS to provide pH‐sensitive release of DOX in cancer therapy; CNTs can be noncovalently wrapped with CS and then loaded with DOX. Due to the deprotonation form of CS, DOX release does not occur at physiological pH, whereas protonation of CS at pH 5–6.5 leads to DOX release due to charge–charge repulsion between CS and DOX, resulting in controlled drug release^267^ that is beneficial for cancer therapy. Another experiment prepared graphene and CNTs for co‐delivery of DOX and paclitaxel (PTX). For improving loading efficiency and release features, further functionalization by CS has been performed. Furthermore, CS provides pH‐sensitive manner release of DOX and PTX and slow release of these antitumor drugs promotes tumor‐suppressor activity.^268^ Based on these experiments, carbon‐based NPs can provide delivery of DOX in cancer therapy and CS modification improves their beneficial features.^99^, ^269^, ^270^, ^271^, ^272^, ^273^, ^274^, ^275^ Figure 7 demonstrates modification of carbon‐based nanomaterials with CS and then, conjugation of folic acid as ligand on nanocarriers to mediate their internalization in cervical cancer cells via endocytosis, resulting in a significant increase in accumulation of DOX in tumor cells.^253^, ^276^, ^277^ Table 3 provides a summary of various nanoparticles modified by CS for purpose of DOX delivery in cancer suppression.

**TABLE 3:** A summary of CS‐based nanostructures for DOX delivery in cancer suppression

Nanostructure	Particle size (nm); zeta potential (mV); encapsulation efficiency or drug loading (%)	Cancer type	In vitro/in vivo	Cell line/animal model	Remarks	Referencs
Estrogen‐functionalized CS nanoparticles	198.2 and 206.4 nm; 28.3 and 30.6 mV; up to 66.33%	Breast cancer	In vitro	MCF‐7 cells	High biocompatibility and antineoplastic activity	278
CS‐raloxifene nanoparticles	26.85 and 34.75 nm; 0.17 and −0.49 mV; up to 98%	Breast cancer	In vitro	MCF‐7 cells	Decreasing proliferation rate by 60% Nanoparticles inhibit cancer progression via suppressing estrogen receptor	279
DOX‐loaded LGCC NPs	200 nm; 20–35 mV; up to 86.4%	Breast and liver cancers	In vitro; in vivo	QGY‐7703 and 4 T1 cells H22 hepatocarcinoma model	Penetrating directly via cell membrane and circumventing endocytic vesicles Cargo release under high GSH levels Endosomal and lysosomal escape High nuclear distribution	262
Catechol‐modified CS‐hyaluronic acid nanoparticles	160 nm; −19.8 mV	Oral cancer	In vitro	HN22 cells	Negative charge and spherical shape High mucoadhesive ability Prolonged release of DOX Reducing cancer proliferation	280
Ethyl cellulose/CS/g‐C_3_N_4_/MoS_2_ core–shell nanofibers	285–370 nm	Breast and cervical cancers	In vitro	MCF‐7 and HeLa cells	Sustained delivery of DOX Inducing cell death up to 89% and 85% in MCF‐7 and HeLa cells, respectively in 7 days	281
Aptamer‐functionalized CS‐bases silica nanostructures	87 nm; 35.9 to −32.3 mV	Breast cancer	In vitro; in vivo	MCF‐7 and 4 T1 cells C26 tumor‐bearing mice	Enhanced cellular uptake Targeted delivery of DOX and anti‐miRNA‐21 in cancer suppression	282
PEGylated CS nanoparticles	169–192 nm; up to 43 mV	Breast cancer	In vitro	MCF‐7 cells	Functionalization of CS nanoparticles with anti‐hMAM and anti‐HER2 promotes selectivity toward cancer cells Exerting dose‐dependent toxicity against cancer cells	283
CMC/PCL nanofibers	300 nm; higher than −30 mV; 90%	Breast cancer	In vitro	MCF‐7 cells	Lack of initial burst release Sustained release for 7 and 25 days Cytotoxicity against tumor cells up to 85%	284
HMSN grafted with CS‐copper sulfide composites	150 nm; −19.6 mV; 46.1%	Breast cancer	In vitro; in vivo	MDA‐MB‐231 cells Mouse model of breast cancer	High biocompatibility Increased cellular uptake by cancer cells Apoptosis induction Increasing survival of mice	285
CS‐, PEG‐ and PVA‐modified MgFe_2_O_4_ ferrite magnetic nanoparticles	78–140 nm; below −21 mV	Breast and colorectal cancers	In vitro	Caco‐2 and SKBR‐3 cells	Decreasing cancer cell viability in a concentration‐dependent manner pH‐sensitive release of DOX 85.86% release of DOX after 72 h	286
CS hydrogel beads	13.5 mV	Breast cancer	In vitro	MCF‐7 cells	High swelling rate (426%) and drug release (81.33% in 144 h) at pH of 5.8 High biocompatibility Decreasing proliferation rate of MCF‐7 cells	287
CMCS/MAGG hydrogel	‐	Breast cancer	In vitro	MCF‐7 cells	pH‐responsive swelling of hydrogels 67.06% release of DOX after 5 days in pH of 5.5 32.13% release of DOX at pH of 7.4 High biocompatibility Cytotoxicity against MCF‐7 cells	287

Abbreviations: CMC, *N*‐carboxymethyl chitosan; CS, chitosan; DOX, doxorubicin; GSH, glutathione; HMSN, hollow mesoporous silica nanoparticle; NPs, nanoparticles; PCL, poly(ε‐caprolactone); PVA, polyvinyl alcohol, PEG, polyethylene glycol.

**FIGURE 8:**
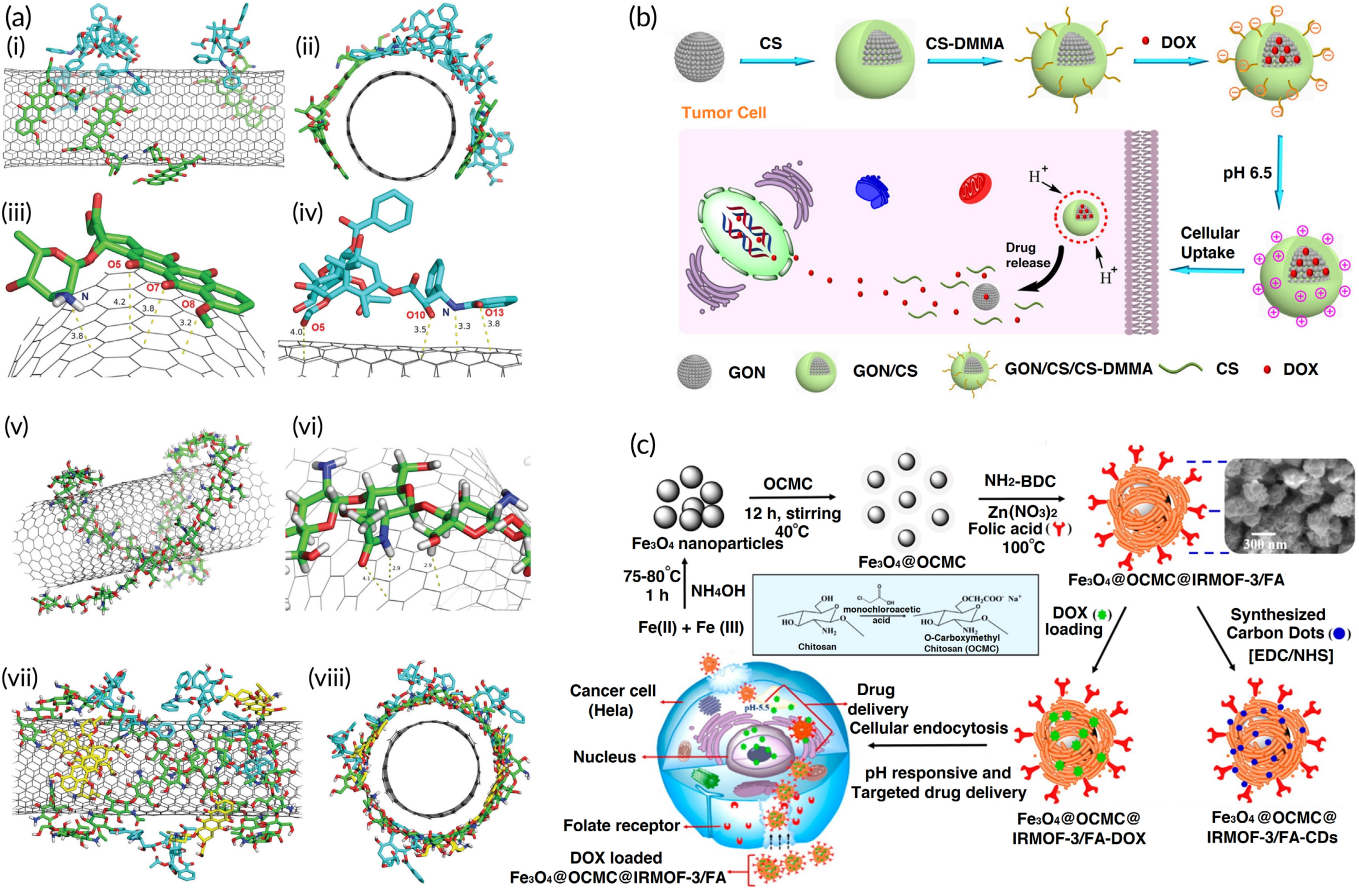
Modification of various kinds of carbon‐based nanostructures with CS for DOX delivery and cancer suppression. (a) Surface modification of single‐walled carbon nanotubes (SWCNs) with CS followed by loading doxorubicin (DOX) and paclitaxel (PTX); (i and ii) DOX and PTX loading on the bare SWCNs from front and side views; a close view of DOX (iii) and PTX (iv) orientation on the side of SWCNs; CS modification on the surface of SWCNs (v and vi); the final drug‐loaded CS‐coated SWCNs from front and side views (vii and viii). *Source*: Reprinted from Ref 277 with permission from RSC. (b) A schematic on the synthesis and applicability of ternary DOX‐loaded graphene oxide nanoparticles (GON)‐CS‐dimethylmaleic anhydride (DMMA) for cancer therapy. *Source*: Reprinted from Ref 276 with permission from ACS publication. (c) A schematic on the synthesis of folic acid (FA)‐anchored *O*‐carboxymethyl CS (OCMC)‐Fe_3_O_4_ modified with carbon dots (CDs) for DOX delivery to the cancer cells. *Source*: Reprinted from Ref 253 with permission from ACS publication. NH_2_‐H_2_BDC, 2‐amino terepthalic acid; IRMOF‐3, metal organic framework

Taking everything together, these studies highlight the fact that various kinds of lipid‐, carbon‐ and metal‐based nanostructures can be modified with CS in improving their characteristics. The nanoparticles demonstrate low particle size, sustained drug release, and high encapsulation efficiency. Furthermore, CS modification may significantly enhance biocompatibility and stability of nanocarriers. Modification of CS‐based nano‐scale delivery systems with ligands such as HA and folic acid increases their selectivity toward tumor cells for specific accumulation of DOX. The CS modification appears to be vital for metal‐ and carbon‐based nanocarriers, as they may show toxicity toward normal cells and such modification improves their biocompatibility (Figure 8).^278^, ^279^, ^280^, ^281^, ^282^, ^283^, ^284^, ^285^, ^286^, ^287^

### 4.8 Directions for clinical application

The current review article demonstrated that rational integration of engineering and biology appears to be promising in treatment of cancer. The gold standard for improving prognosis and survival of cancer patients is chemotherapy. However, chemotherapy failure is a common outcome in cancer patients due to drug resistance. Therefore, there is an urgent need toward development of targeted delivery systems for chemotherapeutic agents. Since DOX is frequently used in clinic for treatment of cancer patients, resistance to its anticancer activities is common. Although problem (drug resistance) is obvious and one of solutions is application of nanocarriers, there are still a number of challenges for use of DOX‐loaded nanostructures in clinical course. The first problem is related to biocompatibility of nanoparticles for DOX delivery in cancer patients. The second problem is affordability and final difficulty is related to large‐scale production of nanocarriers. All of these problems can be solved using greener modification of nanoparticles. Throughout this review article, it was shown that surface modification of various nanocarriers by CS promotes their biocompatibility, safety profile and increases their stability. Furthermore, CS is a natural product that is affordable and can be used for synthesis on larger scale. A number of clinical studies (clinicaltrials.gov) have been conducted on CS application in patients; however, there is no experiment pertaining to the use of CS‐based nanocarriers for DOX delivery in treatment of cancer patients that exploits the afore mentioned benefits of CS. Hopefully, it will occur in the near future.

## 5 CONCLUSION AND REMARKS

The CS is among the most abundant polysaccharides that have received much attention in recent years and a large number of experiments have been performed in revealing therapeutic potentials of CS and its efficacy in NPs synthesis and their modification. Furthermore, as CS is a nature‐derived agent, it is affordable and can be utilized in both preclinical and clinical studies. Regardless of the protective impacts of CS in diabetes, anti‐inflammatory diseases and cardiovascular diseases among others, CS appliance in cancer has undergone a surge due to its cytotoxicity against tumor cells and ability in nanostructure preparation with high biocompatibility. On the other hand, cancer treatment depends on overcoming drug resistance to improve chemotherapy efficacy in tumor cell suppression. Among various chemotherapeutic agents, DOX is a well‐known compound and that is why DOX it has been discussed in this review article. Frequent application of DOX leads to drug resistance and as DOX is an FDA‐approved agent and is utilized in the clinic for the treatment of cancer patients, efforts should be made in reversing this resistance. The present review focused on CS‐NPs for DOX delivery in cancer treatment. Different kinds of stimuli‐responsive CS‐based nanostructures have been developed for DOX delivery including pH‐, redox‐, thermo‐ and multi‐sensitive nanocarriers. The pH of the tumor microenvironment tends to be acidic and is lower than physiological pH. Therefore, pH‐sensitive CS‐based NPs can deliver DOX at tumor site. Due to glycolysis and high proliferation rate of tumor cells, redox‐sensitive NPs are also of importance and GSH levels can induce DOX release from CS nanostructures. The thermosensitive CS‐based hydrogels have been also developed that can be transformed to solid gel at body temperature and provide sustained release of DOX that is of importance for enhancing cytotoxicity against cancer cells and preventing the drug resistance development.

Based on the documented experiments, CS has antitumor activity and it can exert a synergistic impact with DOX. Furthermore, CS‐based NPs promote DOX internalization in cancer cells. The enhanced cellular uptake is vital for promoting the sensitivity of cancer cells to DOX chemotherapy. Furthermore, using other agents such as TPGS with CS can prevent P‐gp activity in inhibiting DOX efflux from tumor cells. Hence, CS‐based NPs can prevent the development of DOX resistance. Notably, CS‐based NPs can provide co‐delivery of DOX with antitumor drugs and nucleic acid therapeutics. This strategy can induce apoptosis in tumor cells in providing DOX sensitivity. Furthermore, the proliferation rate and the invasion of tumor cells undergo a decrease, and the pathway is paved for DOX to induce its tumor‐suppressor activity. Besides, various kinds of NPs including carbon‐, lipid‐, polymer‐ and metal‐based nanostructures can be modified with CS to improve their stability and biocompatibility and provide conditions for DOX complex formation. A search at clinicaltrials.gov shows that CS is currently applied in the clinical course for the treatment of various cancer such as prostate and breast cancers (NCT03202446; NCT03712371). Therefore, future experiments can be directed toward using DOX‐loaded CS‐based nanoarchitectures in the clinic. The application of some of the CS‐modified nanoparticles in clinical course still needs extensive investigation. For instance, CS‐modified metal nanostructures still require examination in terms of biocompatibility, as metal nanoparticles are toxic toward normal cells. The same explanation can be provided for carbon‐based nanomaterials. However, lipid‐based nanoparticles have demonstrated long‐term biocompatibility, especially liposomes and their modification with CS not only increases their biocompatibility but also improves drug release profile and their stability, paving the way for clinical application.

## CONFLICT OF INTERESTS

The authors declare no conflict of interest.

### PEER REVIEW

The peer review history for this article is available at https://publons.com/publon/10.1002/btm2.10325.

## Data Availability

Data sharing is not applicable to this article as no new data were created or analyzed.
